# Classical synchronization indicates persistent entanglement in isolated quantum systems

**DOI:** 10.1038/ncomms14829

**Published:** 2017-04-12

**Authors:** Dirk Witthaut, Sandro Wimberger, Raffaella Burioni, Marc Timme

**Affiliations:** 1 Forschungszentrum Jülich, Institute for Energy and Climate Research (IEK-STE), 52428 Jülich, Germany; 2 Institute for Theoretical Physics, University of Cologne, Zuelpicher Str. 77, 50937 Köln, Germany; 3 Network Dynamics, Max Planck Institute for Dynamics and Self-Organization (MPIDS), Am Faßberg, 37077 Göttingen, Germany; 4 Dipartimento di Scienze Matematiche, Fisiche ed Informatiche, Universitá di Parma, Via G.P. Usberti 7/a, 43124 Parma, Italy; 5 INFN, Sezione di Milano Bicocca, Gruppo Collegato di Parma, Parco Area delle Scienze, 7/A, 43124 Parma, Italy; 6 Department of Physics, University of Darmstadt, 64289 Darmstadt, Germany; 7 Institute for Theoretical Physics, Technical University of Dresden, 01062 Dresden, Germany

## Abstract

Synchronization and entanglement constitute fundamental collective phenomena in multi-unit classical and quantum systems, respectively, both equally implying coordinated system states. Here, we present a direct link for a class of isolated quantum many-body systems, demonstrating that synchronization emerges as an intrinsic system feature. Intriguingly, quantum coherence and entanglement arise persistently through the same transition as synchronization. This direct link between classical and quantum cooperative phenomena may further our understanding of strongly correlated quantum systems and can be readily observed in state-of-the-art experiments, for example, with ultracold atoms.

## Abstract (web summary)

Collective phenomena in many-body systems include synchronization in classical and entanglement in quantum systems. Here the authors study isolated many-body quantum systems and demonstrate that synchronization emerges intrinsically, accompanied by the onset of quantum coherence and persistent entanglement.

Understanding collective dynamical phenomena constitutes a topical challenge across physics and beyond, with distinct implications for the classical and quantum realms. How collective phenomena in classical and quantum worlds are linked is largely unknown. Synchronization constitutes one of the most basic cooperative dynamics in classical systems. It indicates the locking of states of coupled classical units and governs the dynamics of physical, chemical, and biological systems^1^,^2^,^3^,^4^,^5^,^6^,^7^,^8^. Entanglement constitutes the most fundamental phenomenon in many-body quantum systems and indicates correlations that are genuinely quantum mechanical. Two quantum particles are entangled if they cannot be described by independent single-particle states. Such entanglement thereby determines the quantum systems' inherent complexity^9^,^10^ and unique computational power^11^,^12^.

In this article, we present a direct link between classical synchronization and quantum entanglement. We investigate a paradigmatic class of isolated nonlinearly coupled quantum systems combining the classical theory of synchronization with simulations of quantum dynamics and mean-field as well as higher-order analysis. We reveal that and how synchronization phenomena impact entanglement. Intriguingly, transient squeezing and number fluctuations indicating genuine entanglement emerge through and exactly at the transition to classical synchronization. Moreover, the dynamics of classical phase locking quantitatively predicts the growth of quantum number fluctuations, and for large system sizes becomes an exact indicator of the growth. As the quantum system is isolated, synchronization is not externally induced but emerges through self-organized dynamics. We demonstrate how this quantum-classical link on the level of collective phenomena may be experimentally verified, for example with ultracold atoms^13^,^14^,^15^,^16^,^17^. For a paradigmatic and experimentally relevant class of systems, these results thus indicate that the substantial parts of the emergence of entanglement—a genuine quantum feature—can be traced back to a classical synchronization process.

## Results

### Signatures of synchronization

Consider the dynamics of a quantum many-body system described by Schrödinger's equation 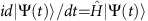 with the Hamiltonian


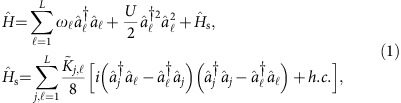


describing *L* spatially localized modes *j*∈{1, …, *L*} (ref. 18) with on-site two-body interactions of energy scale *U*. 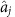 denotes the annihilation and 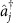 the creation operator for the *j*th mode and 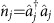 is the number operator.

**Figure 1:**
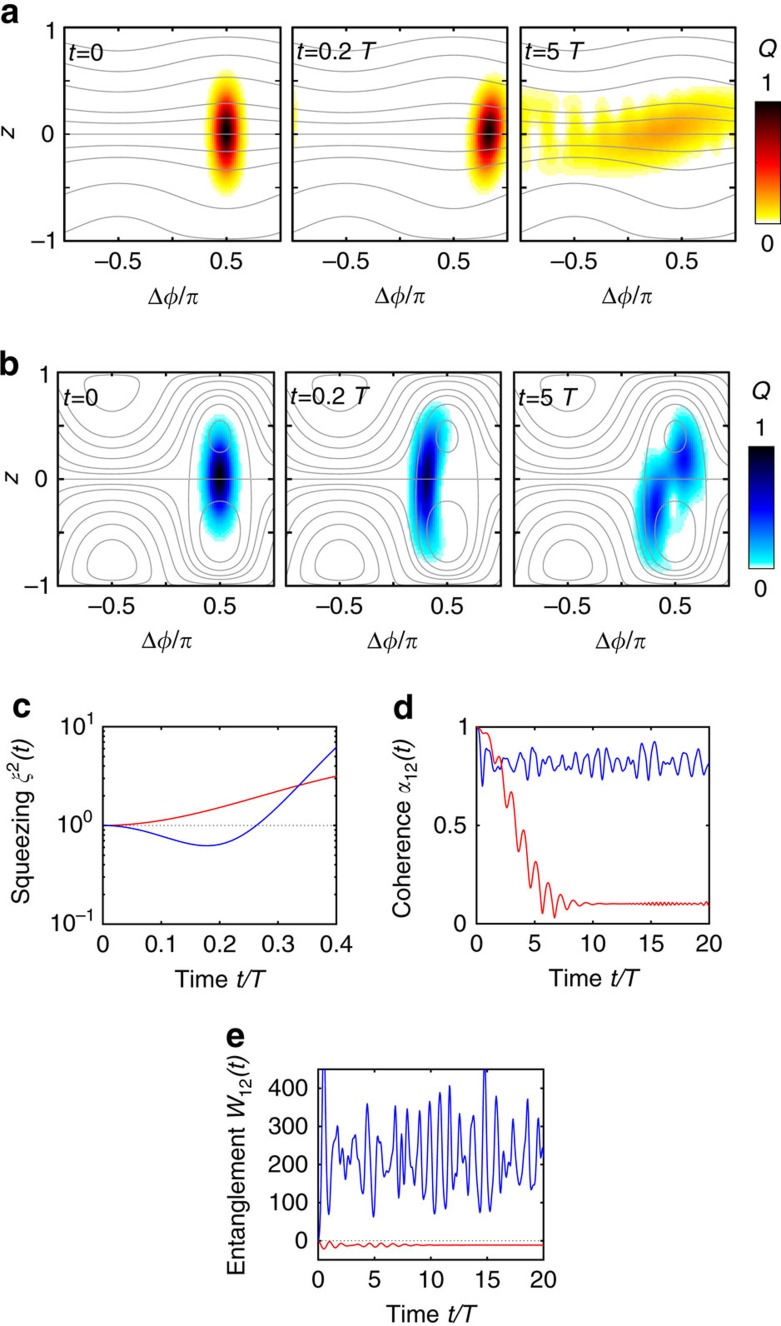
From short-term quantum squeezing to long-term phase coherence and number entanglement. Quantum dynamics of two coupled oscillators or modes; the initial state being a two-mode coherent state |*z*, Δ*φ*〉=|0, *π*/2〉. (**a**) For sufficiently small coupling strengths (here *K*=0.1*ω*) the two modes simply dephase. The Husimi density *Q*(*z*, Δ*φ*; *t*)≡|〈*z*, Δ*φ*|*Ψ*(*t*)〉|^2^ (ref. 43) spreads out along the phase direction. (**b**) Squeezing and number entanglement emerge for a sufficiently large coupling strength (here *K*=0.8*ω*). At intermediate times, the Husimi density is *Q*(*z*, Δ*φ*; *t*) is compressed in the phase direction. For long times, the quantum state is trapped in the right half of phase space implying preservation of phase coherence. The grey lines in (**a**,**b**) show trajectories of the classical mean-field system (2) bearing the Kuramoto model. (**c**–**e**) Evolution of (**c**), the squeezing parameter *ξ*^2^ (refs 13, 19), (**d**) the phase coherence *α*_12_ and (**e**) the number entanglement *W*_12_ for *K*=0.8*ω* (blue line) and *K*=0.1*ω* (red line). Short-term quantum squeezing, long-term phase coherence, and number entanglement are observed for a strong coupling *K*=0.8*ω*. Parameters for all panels are *N*=40, *U*=0.4/*N*, *T*=2*π*/*ω* and 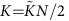. The initial state is a two-mode coherent state |*z*, Δ*φ*〉=|0, *π*/2〉.

The quantum many-body system (1) exhibits a sharp transition from a weakly to a strongly correlated regime. When the coupling strengths 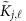 exceed a critical value, correlations emerge dynamically and persist independent of the initial state. This transition (Fig. 1) becomes apparent already for systems with just two modes, which arise in the longitudinal Lipkin-Meshkow-Glick model (see Supplementary Note 1). Figure 1a,b illustrate the different dynamical regimes for a coherent initial state |*Ψ*(0)〉=|*z*, Δ*φ*〉, that is, a state which is maximally localized in phase space (see Methods section).

For small coupling strengths 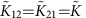, correlations remain negligible and the modes gradually dephase, so the phase coherence *α*_12_(*t*) defined by 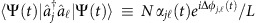 decays rapidly. The Husimi function *Q*(*t*), representing the quantum phase space density (see Methods section), spreads in the phase direction, such that the relative phase of the two modes becomes undefined.

In contrast, for sufficiently large coupling strengths 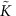, we observe transient squeezing of the quantum state (Fig. 1c): The phase space density *Q*(*t*) is compressed in the phase direction and the uncertainty of the relative phase decreases below the standard uncertainty limit, indicating many-body entanglement^13^,^19^. Moreover, a strong phase coherence prevails in the long term (Fig. 1d). The reduction of phase fluctuations is accompanied by the emergence of number entanglement: The number fluctuations exceed the maximum possible for any separable (non-entangled) quantum state, indicated by the entanglement parameter *W*_12_>0 in Fig. 1e (see Methods section). Strikingly, this type of entanglement is persistent.

**Figure 2:**
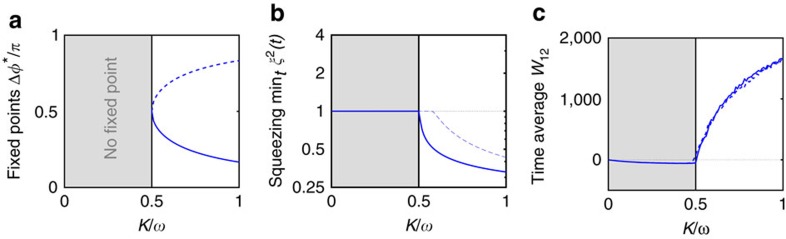
Classical synchronization indicates quantum squeezing and number entanglement. We consider two coupled oscillators, respectively, two quantum modes with variable coupling strength *K*. (**a**) The fixed points of the Kuramoto model (4). A stable (solid) and an unstable (dashed) fixed point emerge in a saddle node bifurcation at *K*_c_=|*ω*|/2. (**b**) The temporal minimum of the squeezing parameter 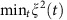 (refs 13, 19) as a function of the coupling strength *K*. For *U*=0 (solid line) the transition to quantum squeezing is located at the critical coupling for classical synchronization, *K*_c_=*ω*/2. The on-site interaction term ∼*U* suppresses squeezing such that the transition occurs at a higher value of *K* for *U*=0.4/*N* (dashed line). (**c**) Long-time average of the entanglement parameter *W*_12_ as a function of the coupling strength *K*. Persistent entanglement *W*_12_>0 (see ref. 50 and Methods) emerges with classical synchronization at *K*>*K*_c_=*ω*/2, independent of *U*. The initial quantum state is a two-mode coherent state |*z*, Δ*φ*〉=|0, *π*/2〉 with *N*=100 and *ω*=1 in all cases.

Squeezing and entanglement systematically emerge for coupling strengths above some critical value, whereas they are absent below, see Fig. 2. The observed transition indicates a quantum analogue of the classical synchronization transition^1^,^2^,^3^,^4^. To see this, consider the mean-field limit and derive the equations of motion for the amplitudes 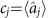 from Heisenberg's equation, neglecting quantum fluctuations and approximating 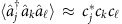. We obtain


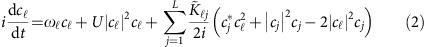


(see Supplementary Note 3 for more details). Given a total number *N* of excitations, the manifold of the phase space 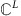 defined by 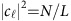 for all 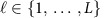 is invariant under the dynamics such that 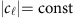 in time (see Supplementary Note 3 and ref. 20). With initial conditions on this manifold denoted by 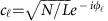 the dynamics (2) reduces to


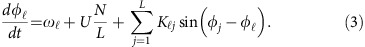


The intrinsic frequencies and rescaled coupling strengths become 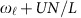 and 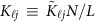, respectively.

This mean field limit constitutes a system of Kuramoto oscillators^2^—a paradigmatic model of classical nonlinear dynamics characterizing synchronization and other collective phenomena^3^,^4^. For two modes, the dynamics is fully characterized by the phase difference Δ*φ*=*φ*_2_−*φ*_1_ and the population imbalance *z*=(|*c*_2_|^2^−|*c*_1_|^2^)/(|*c*_2_|^2^+|*c*_1_|^2^) as the total number of excitations is conserved. The phase dynamics on the invariant manifold *z*=0 becomes


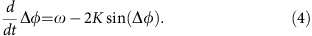


with *ω*=*ω*_2_−*ω*_1_. This Kuramoto system bifurcates at *K*_c_=|*ω*|/2, precisely indicating the quantum transition point, see Fig. 2. Below *K*_c_ no steady states exist and the phases are unlocked. For *K*>*K*_c_ phase locking emerges such that Δ*φ*(*t*) tends to the fixed point Δφ*=arcsin(*ω*/2*K*), which shapes the corresponding quantum dynamics (Fig. 1a versus b): The Husimi function is contracted at the fixed point such that phase squeezing emerges. Simultaneously, the dynamics is unstable in the *z*-direction, indicating the growth of number fluctuations. The classical Kuramoto dynamics can thus be seen as a skeleton of the full quantum dynamics^21^ and the onset of classical synchronization as a skeleton for the emergence of quantum correlations.

**Figure 3:**
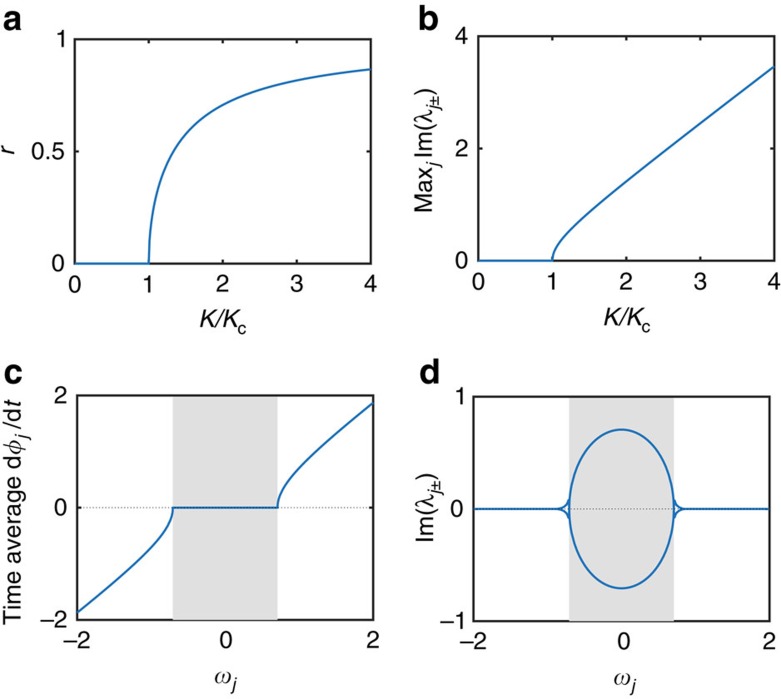
Classical phase locking indicates growth of quantum number fluctuations. (**a**) The classical synchronization order parameter *r* measures the degree of locking emerging for *K*>*K*_c_. (**b**) Synchronization implies an exponential growth of quantum fluctuations. The maximum growth rate 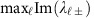 in the limit of large systems becomes proportional to the classical synchronization order parameter *r* as predicted by equation (11). (**c**) The average phase velocity 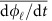 in the Kuramoto model as a function of natural frequency 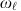. Oscillators in the grey region are phase locked, that is, the average phase velocity is identical. (**d**) Quantum number fluctuations grow rapidly for the oscillators in the the region of classical phase locking (grey), indicated by non-zero values of the growth rate 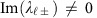. Results are shown for globally coupled oscillators, that is, 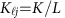 in the limit *L*→∞. Natural frequencies are drawn from a Lorentzian distribution *g*(*ω*), for which *r*(*K*)=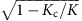 near *K*_c_=2/(*πg*(0)) (refs 2, 3). Parameters are *U*=0 and *K*=2*K*_c_ in **c**,**d**.

The correspondence of classical phase locking and the growth of quantum fluctuations becomes analytically exact for large populations of globally coupled oscillators, that is, 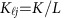 with large 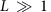 (Fig. 3a–d). We define the Kuramoto order parameter^1^,^3^,^4^


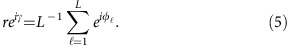


In the generic case, the magnitude *r* relaxes to a fixed value measuring the degree of phase order and *γ* oscillates with the mean frequency 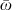
+*Uν*, *ν*=*N*/*L* being the density of atoms or excitations per mode. Transforming to a co-rotating frame of reference, *γ* becomes constant and the classical equations of motion (3) simplify to


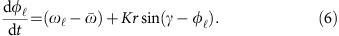


A bifurcation occurs when the coupling *K* increases: For *K*⩽*K*_c_ all oscillators drift independently such that *r*=0. For *K*>*K*_c_ the oscillators with 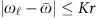 get phase-locked, 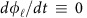 such that *r*>0.

To describe quantum fluctuations beyond mean-field, we decompose the annihilation operators into the condensate mode 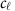 and the quantum fluctuations 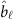, 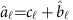 and insert this ansatz into the Heisenberg equations of motion (see Supplementary Note 4 and Supplementary Fig. 4 for more details). To linear order in 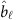 this yields the Bogoliubov-de Gennes equations^22^


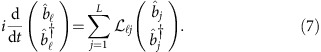


On the Kuramoto manifold the Bogoliubov-de Gennes operator is given by


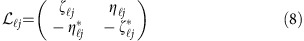


where the coefficients are given by


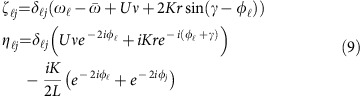


in the rotating frame. In the limit *N*, *L*→∞ with fixed *ν* = *N*/*L*, all terms with 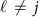 vanish as *L*^−1^ such that the operator 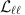 describes whether quantum fluctuations grow. For *r*=0 all eigenvalues 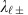 of 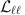 are real, implying that fluctuations do not grow. Once synchronization sets in and *r*>0, the eigenvalues of the phase-locked oscillators are purely imaginary,


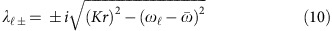


such that quantum fluctuations grow exponentially as 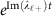. The maximum growth rate becomes


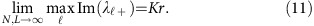


This growth rate scales as the classical synchronization order parameter (Fig. 3b). Drifting oscillators typically have real eigenvalues, except for the ones in the immediate vicinity of the phase-locked region (compare Fig. 3c versus d). Hence the classical synchronization transition^1^,^2^,^3^,^4^,^5^ has a direct quantum counterpart.

### Potential experimental realizations

**Figure 4:**
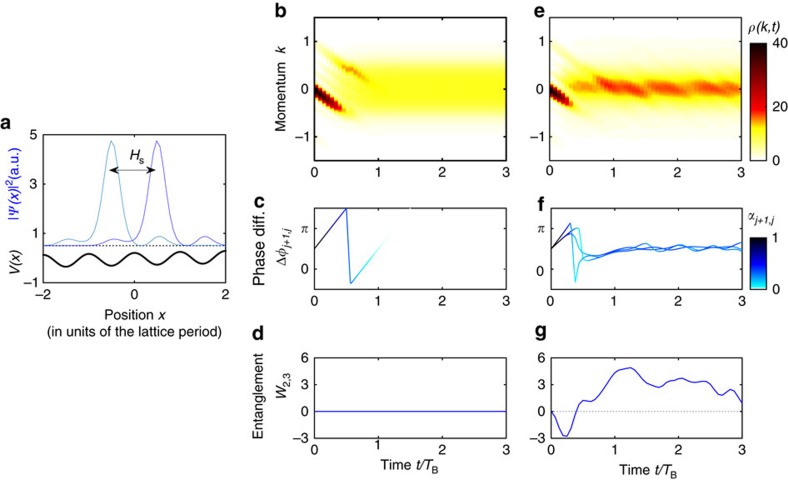
Quantum synchronization manifests in momentum space localization for ultracold atoms. (**a**) In a tilted or accelerated optical lattice, the single-particle eigenstates (blue lines) are localized in a few wells of the lattice potential (bold black line). The overlap with other wells induces a nonlinear coupling between the states described by 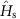 (**b**,**c**) Without coupling 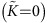, the modes dephase due to the on-site interaction ∼*U*. The coherences *α*_*j*,*j*+1_ decay to zero and the momentum density distribution *ρ*(*k*, *t*) spreads over the entire Brillouin zone. (**d**) For 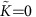 there is no entanglement, *W*_2,3_=0. (**e**,**f**) For 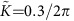, the nonlinear coupling leads to phase locking at *φ*_*j*+1_−*φ*_*j*_≈*π*/2 at intermediate values of the coherence *α*_*j*,*j*+1_. This leads to a localization of the momentum space density *ρ*(*k*, *t*) around *k*=0, which can be readily detected in a time-of-flight image. (**g**) Synchronization implies strong persistent many-body entanglement quantified by the entanglement parameter *W*_2,3_>0 (see ref. 50 and Methods). Parameters are *L*=4, *N*=12 and *U*=1.2/2*π* in units of *ω*_B_=2*π*/*T*_B_. The initial state 
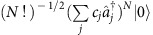
 is a pure Bose-Einstein Condensate (BEC) with zero momentum.

Our predictions are observable in experiments, for example, with Bose-Einstein condensates (BECs) in optical lattices^13^,^14^,^15^,^16^,^17^ or modulated photonic lattices^23^,^24^. In a tilted or accelerated lattice, the eigenmodes are localized (Fig. 4a) and the energies are arranged in a ladder, 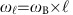 (ref. 25). The atomic interactions induce the nonlinear coupling 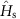 of the neighbouring modes with 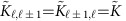 and 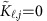 otherwise. A detailed discussion of how these parameters depend on the experimental setting is provided in the Supplementary Note 2 and the Supplementary Figs 1–3.

Synchronization is detected from the momentum density *ρ*(*k*, *t*) measured in a time-of-flight image (Fig. 4b). For weak coupling 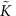, the modes dephase^15^ such that the coherences 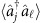 vanish. No relative phase is defined and *ρ*(*k*, *t*) delocalizes over the Brillouin zone (Fig. 4b,c). Strong coupling induces synchronization such that the coherences are partly preserved and *ρ*(*k*, *t*) shows a localized peak which does not blur (Fig. 4e,f). The peak remains steady in the center of the Brillouin zone as the phases are locked at a constant value (Fig. 4d). Momentum space localization thus provides a robust experimental quantum signature of synchronization. Synchronization implies number fluctuations signalling entanglement (Fig. 4g), as above for two modes. This entanglement is persistent and emerges for all pure BECs and Fock states with homogeneous density.

Another possibility to experimentally realize our predictions is given by Floquet engineered optical lattices^16^,^17^.

### Robustness to dissipation

In many models studied so far, quantum signatures of synchronization are induced externally, via dissipation or a common driving^26^,^27^,^28^,^29^,^30^,^31^,^32^,^33^,^34^,^35^. In contrast, the coupling to the environment is not the cause of synchronization in the quantum many-body system (1), which directly bears the Kuramoto model in the mean-field limit. Indeed, this classical synchronization model qualitatively predicts that the quantum system relaxes to different states depending on the coupling strength. The phase coherence 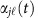, which is most easily accessible in experiments, converges to a non-zero value up to some small residual fluctuations (Figs 1 and 4). But how robust is this intrinsic form of quantum synchronization to perturbations from the environment?

**Figure 5:**
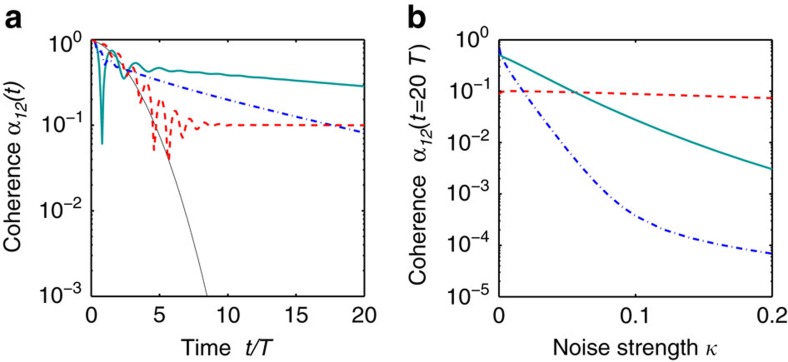
Synchronization-induced phase coherence is robust to noise. (**a**) Without coupling (*K*=0, thin black line), the coherence *α*_12_(*t*) of two coupled modes decays rapidly in the presence of phase noise. The synchronisation coupling 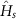 slows down this decay by orders of magnitude (*K*=0.8 dash-dotted blue line, *K*=0.4 solid turquois line). For a weak but non-zero coupling, the phase coherence becomes almost constant in time at a value of *α*_12_(*t*)≈10^−1^ after a transient decrease (*K*=0.1 dashed red line). The noise strength is *κ*=0.02. (**b**) Phase coherence is remarkably robust especially in the subcritical regime 0<*K*⩽*K*_c_. For *K*=0.1 the coherence *α*_12_(*t*) at time *t*=20 *T* is almost independent of the noise strength *κ*. The remaining parameters are as in Fig. 1.

We are reporting a particular destructive case of quantum dissipation, where independent phase noise couples to all modes of the system. Such a noise source arises in experiments with ultracold atoms in optical lattices due to incoherent scattering of photons from the lattice beams^36^ or collisions with the background gas^37^. The noisy dynamics can be well captured using a quantum master equation in Lindblad form


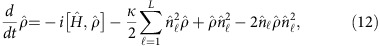


where 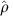 is the density operator and *κ* the noise rate. Numerical simulations of the master equation (12) for two modes indicate the influence of phase noise on the evolution of phase coherence. Without the synchronization coupling (for *K*=0), already weak noise completely destroys the phase coherence *α*_12_(*t*) within a few periods *T* as shown in Fig. 5. In contrast, the decay of *α*_12_(*t*) is slower by orders of magnitude in the presence of the coupling (for *K*>0). Hence, the effects described in the present paper should be experimentally observable also in the presence of noise.

The robustness of the phase coherence depends crucially on the coupling strength *K*. For supercritical coupling *K*>*K*_c_ the quantum state tends to a highly entangled superposition of atoms being localized in one of the wells, making it more susceptible to noise. Phase coherence decays in time, but the decay is still much slower than without coupling. The mode-coupling through the Hamiltonian 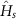 induces phase coherence also for 0<*K*⩽*K*_c_, but without strong number fluctuations. This form of coherence is remarkably robust. After an initial drop, the phase coherence *α*_12_(*t*) remains almost constant in time for the subcritical coupling *K*=0.1 (Fig. 5a) and the final value of *α*_12_(*t*) is almost independent of the noise strength *κ* (Fig. 5b). For different sources of noise or dissipation (less uncorrelated for instance) we expect an even better robustness in all mentioned regimes.

## Discussion

In summary, we have unearthed the manifestation of classical synchronization in a class of quantum many-body systems, providing a direct link between collective classical and quantum dynamics. So far, synchronization and entanglement have been mostly studied as two separate phenomena in the classical and quantum worlds, respectively. Recent previous works considered aspects of synchronization in open quantum model systems, where the coupling to the environment is crucial. The interaction with a common thermal bath can incude synchronization of qubits^26^ or harmonic oscillators^27^, as well as a common classical driving field^28^. Synchronization has also been studied for quantum van der Pol oscillators^29^,^30^,^31^,^32^ and other driven dissipative oscillators^33^,^34^. In all these cases, dissipation and external driving play a crucial role, for instance the self-sustained oscillations of the van der Pol oscillators are entirely driven by the exchange of excitations with the bath. It has also been shown that quantum effects can prevent the occurrence of synchronization of coupled spins^38^. The Kuramoto model itself has been recovered in the semiclassical limit of different quantum system, in particular particles moving in tilted washboard potential^35^, coupled optomechanical oscillators^39^ or Josephson junction arrays^40^. In contrast, we here analysed a class of isolated quantum systems, demonstrating that synchronization emerges as an intrinsic system feature. We recover the celebrated Kuramoto model in the mean-field limit, which can be seen as a skeleton for the full-quantum many-body dynamics.

Indeed, the transition to synchronization clearly indicates squeezing, long-term coherence and persistent entanglement. Moreover, the dynamics of phase locking in the synchronization process indicates the growth of number fluctuations, becoming exact in the limit of large system sizes. Our findings can be directly verified by state-of-the-art experiments, for instance with ultracold atoms in accelerated or driven optical lattices^13^,^14^,^15^,^16^,^17^ or modulated photonic lattices^23^,^24^. These experiments are facilitated by the observed robustness with respect to dissipation. They offer a unique control over the system parameters such that various distributions of natural frequencies can be realized which can give rise to different types of synchronization phase transitions^41^,^42^. Advanced imaging techniques allow to observe the global phase coherence as well as number distributions with single site resolution. These results thus offer a novel perspective on a correspondence between classical and quantum dynamics, on the level of collective phenomena.

## Methods

### Coherent states

Spin coherent states are defined as 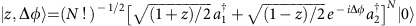. They are maximally localized in phase space and thus provide a natural link to the classical mean-field dynamics^18^. The projection of a quantum state on a coherent states defines the Husimi function *Q*(*z*, Δ*φ*; *t*)≡|〈*z*, Δ*φ*|*Ψ*(*t*)〉|^2^. It carries all information about the quantum state and shares properties of a classical phase space density^43^.

### Squeezing

To quantify squeezing one defines the collective operators 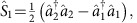 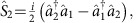 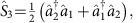 which form an angular momentum algebra. The squeezing parameter is then defined as 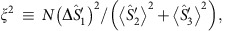 where 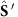 is a rotation of the vector operator 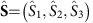. Spectroscopic squeezing with *ξ*^2^<1 is only possible for entangled states and enables high-precision quantum metrology^13^,^19^.

### Number entanglement

The variance of the number difference between two modes 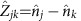 is bounded for every pure separable state as 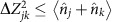 (ref. 44). If the entanglement parameter 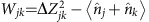 exceeds zero for a pure state, this unambiguously proves the entanglement of the modes. States with large *W*_*jk*_ are used in precision quantum metrology^45^,^46^,^47^.

### Tilted optical lattices

To study the quantum dynamics in tilted or accelerated optical lattices, we expand the bosonic field operator in the single-particle eigenmodes 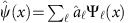 assuming that tunneling to excited Bloch bands is negligible. The eigenmodes 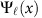 are arranged in ladders with equidistant eigenenergies 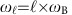, where *ω*_B_ is the Bloch frequency^25^. Depending on the tilting, the eigenmodes are strongly localized in real space (Fig. 4a), such that they couple only to the nearest neighbours. The momentum space density is given by


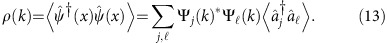


In the non-interacting case *U*=*K*=0 the coherences are constant in magnitude and the phases evolve according to 
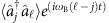
. The atoms show a periodic dynamics, referred to a Bloch oscillations, with the Bloch period *T*_B_=2*π*/*ω*_B_ (refs 25, 48). Pure on-site interactions lead to dephasing such that the coherences vanish, 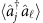
→0 for 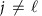 and the momentum density reads 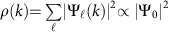, which is extended over the entire first Brillouin zone^15^,^49^.

### Data availability

The data that support the findings of this study (in particular simulation source code and figure raw data) are available from the corresponding author upon request.

## Additional information

**How to cite this article:** Witthaut, D. *et al*. Classical synchronization indicates persistent entanglement in isolated quantum systems. *Nat. Commun.* **8,** 14829 doi: 10.1038/ncomms14829 (2017).

**Publisher's note**: Springer Nature remains neutral with regard to jurisdictional claims in published maps and institutional affiliations.

## Supplementary Material

Supplementary Information Supplementary Figures, Supplementary Notes and Supplementary References.

Peer Review File
