# Supplementary material for: Classical synchronization indicates persistent entanglement in isolated quantum systems
Source: Nat Commun. 2017 Apr 12;8:14829. doi: 10.1038/ncomms14829 (PMC5394286; doi:10.1038/ncomms14829)
Supplement: Supplementary Information — Supplementary Figures, Supplementary Notes and Supplementary References. [file ncomms14829-s1.pdf]

Supplementary Figure 1

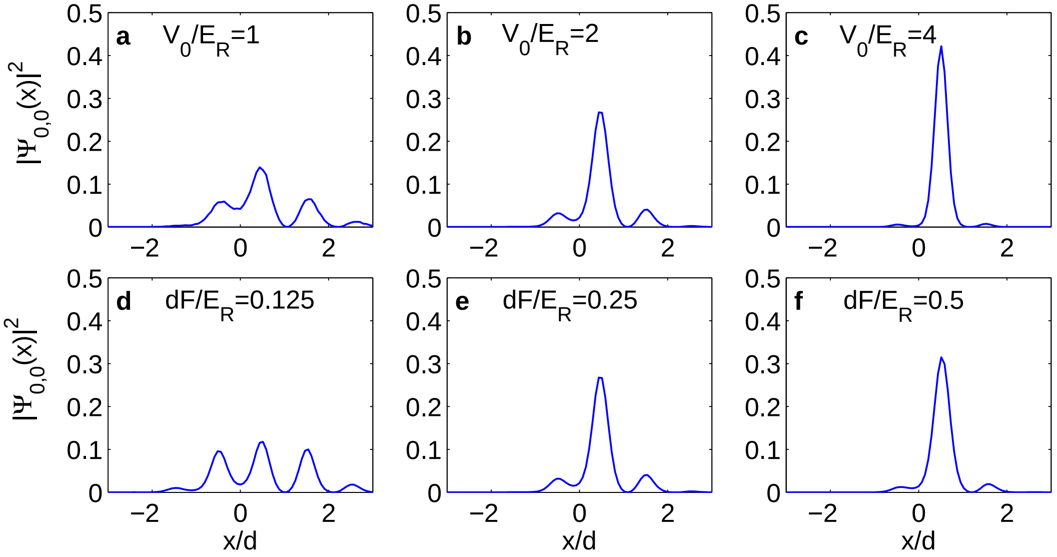

Supplementary Figure 1. **Localization of Wannier-Stark resonance states.** The figure shows the propability density $|\Psi_{0,0}(x)|^2$ for the Wannier-Stark resonance state in the lowest ladder $\alpha = 0$ for different values of the lattice depth V_0 and the external field strength F. **a-c,** The localization of the Wannier-Stark resonance states increases with the lattice depth, shown for **a,** $V_0/E_R = 1$, **b,** $V_0/E_R = 2$ and **c,** $V_0/E_R = 4$. The field strength is $dF/E_R = 0.25$. **d-f,** The localization of the Wannier-Stark resonance states also increases with the strength of the external field or acceleration, shown for **d,** $dF/E_R = 0.125$, **e,** $dF/E_R = 0.25$ and **f,** $V_0/E_R = 0.5$. The lattice depth is $V_0/E_R = 2$ and $E_R = \hbar^2\pi^2/2md^2$ denotes the recoil energy of the lattice photons.

Supplementary Figure 2

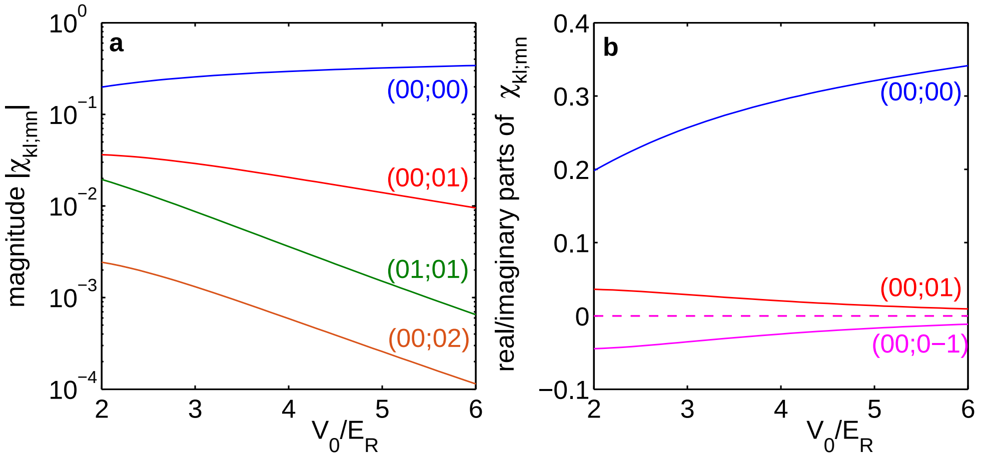

Supplementary Figure 2. **Scaling of Wannier-Stark interaction terms with the lattice depth.** The interaction term in the Wannier-Stark basis is determined by the overlap intergrals $\chi_{k,\ell;m,n} = \int \Psi^*_{0,k}(x)\Psi^*_{0,\ell}(x)\Psi_{0,m}(x)\Psi_{0,n}(x)\,dx$. **a,** Scaling of the magnitude of the overlap integrals with the lattice depth V_0. For a sufficiently deep lattice the terms $\chi_{0,0;0,0}$, $\chi_{0,0;0,+1}$ and $\chi_{0,0;0,-1}$. dominate. All other overlaps are significantly smaller and are neglected in the current analysis. **b,** We use that $\mathrm{Im}(\chi_{0,0;0,\pm1}) \approx 0$ (dashed lines) and $\chi_{0,0;0,+1} \approx -\chi_{0,0;0,-1}$ (solid lines). The field strength is given by $dF/E_R = 0.4$ and $E_R = \hbar^2\pi^2/2md^2$ denotes the recoil energy of the lattice photons.

Supplementary Figure 3

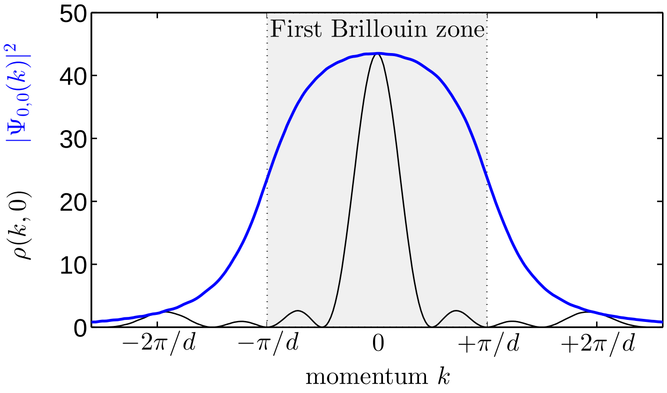

Supplementary Figure 3. **Wannier-Stark resonances in momentum space.** A Wannier-Stark resonance state $|\Psi_{0,0}(k)|^2$ (thick blue line) and the atom density $\rho(k,0)$ (thin black line) for a homogeneous BEC with $c_j = 1/\sqrt{L}$ for $j = 1, \ldots L$. Parameters are $V_0/E_R = 2$, $dF/E_R = 0.25$ and $L = 4$.

Supplementary Figure 4

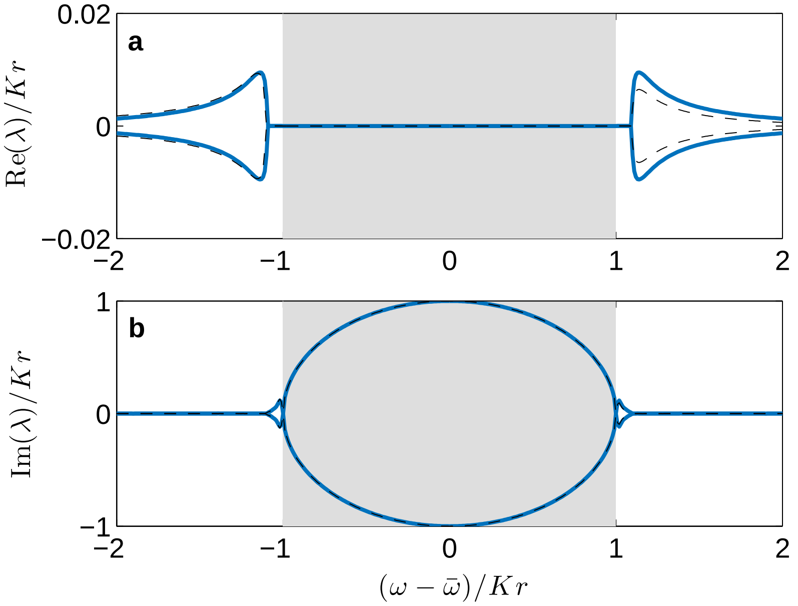

Supplementary Figure 4. Eigenvalues of the Bogoliubov-de Gennes operator for global coupling. The eigenvalues of the Bogoliubov-de Gennes operator $\bar{\mathcal{L}}$ indicate the stability of a Bose-Einstein condensate: The imaginary part gives the growth rate of quantum fluctuations in the respective mode. In the limit of many globally coupled oscillators ($L \to \infty$), for which the quantum dynamics of the oscillators decouples and the eigenvalues essentially depend on the ratio of the natural frequency $\omega - \bar{\omega}$ and the order parameter Kr. Shown is **a,** the real and **b,** the imaginary part of the eigenvalues for $U\nu = 0$ (—) and $U\nu = Kr$ ($- - -$).

Supplementary Note 1

Equivalence to the longitudinal Lipkin-Meshkov-Glick model

We study quantum signatures of synchronization for the many-body Hamiltonian

$$\hat{H} = \sum_{j=1}^{L} \omega_j \hat{a}_j^\dagger \hat{a}_j + \frac{U}{2} \hat{a}_j^{\dagger 2} \hat{a}_j^2 + \hat{H}_\mathrm{s} \tag{1}$$

$$\hat{H}_\mathrm{s} = \sum_{j,\ell=1}^{L} \frac{\widetilde{K}_{j,\ell}}{8} \left[i(\hat{a}_j^\dagger \hat{a}_\ell - \hat{a}_\ell^\dagger \hat{a}_j)(\hat{a}_j^\dagger \hat{a}_j - \hat{a}_\ell^\dagger \hat{a}_\ell) + \mathrm{h.c.} \right].$$

For $L = 2$ oscillators, this Hamiltonian describes the Lipkin-Meshkov-Glick (LMG) model with a longitudinal external field as we show in the following. The LMG model was originally introduced to analyze shape transitions of atomic nuclei [1]. By now, it is a standard model in statistical physics for the study of quantum phase transitions and the quantum-to-classical correspondence [2]. The LMG model describes N indistinguishable particles with 2 internal states, e.g. N hadronic spins or N two-level atoms. The particles interact symmetrically with each other and with an external magnetic field $\mathbf{h}$. The Hamiltonian in its most general form is then given by

$$\hat{H} = \tfrac{1}{N}(\lambda_x \hat{S}_x^2 + \lambda_y \hat{S}_y^2) + h_x \hat{S}_x + h_y \hat{S}_y + h_z \hat{S}_z \,. \tag{2}$$

The $\hat{S}_{x,y,z}$ are the collective spin operators, which form an angular momentum operator with quantum number $N/2$,

$$[\hat{S}_a, \hat{S}_b] = i\epsilon_{abc}\hat{S}_c,$$
$$\hat{S}_x^2 + \hat{S}_y^2 + \hat{S}_z^2 = \frac{N}{2}\left(\frac{N}{2} + 1\right), \tag{3}$$

where ϵ_{abc} is the totally anti-symmetric tensor.

Traditionally, the case of a purely transversal field ($h_{x,y} = 0$) has received the most interest. However, a longitudinal field arises naturally in many quantum-optical realizations of the LMG model [3–5]. We consider the case that λ_x and λ_y have opposite signs and rotate the coordinate system according to

$$\hat{S}_x = \cos(\theta)\hat{S}'_x + \sin(\theta)\hat{S}'_y \tag{4}$$

$$\hat{S}_y = -\sin(\theta)\hat{S}'_x + \cos(\theta)\hat{S}'_y \tag{5}$$

with $\tan(\theta) = \sqrt{-\lambda_y/\lambda_x}$. The LMG Hamiltonian then reads

$$\begin{aligned} H' = & \frac{\lambda_x \cos(\theta)^2 + \lambda_y \sin(\theta)^2}{N} \hat{S}_x'^2 \\ & + \frac{(\lambda_x - \lambda_y)\cos(\theta)\sin(\theta)}{N} (\hat{S}_x' \hat{S}_y' + \hat{S}_y' \hat{S}_x') \\ & + (h_x \cos(\theta) - h_y \sin(\theta)) \hat{S}_x' \\ & + (h_x \sin(\theta) + h_y \cos(\theta)) \hat{S}_y' + h_z \hat{S}_z \end{aligned} \tag{6}$$

up to a constant. Tuning the external field such that

$$\begin{aligned} & h_x \sin(\theta) + h_y \cos(\theta) = 0 \\ & h_z = 0, \end{aligned} \tag{7}$$

and introducing the mapping

$$\begin{aligned} \widetilde{K} = & \frac{(\lambda_x - \lambda_y)\cos(\theta)\sin(\theta)}{N} \\ \omega = & h_x \cos(\theta) - h_y \sin(\theta) \\ U = & \frac{\lambda_x \cos(\theta)^2 + \lambda_y \sin(\theta)^2}{N}, \end{aligned} \tag{8}$$

the LMG Hamiltonian reads

$$H' = \omega \hat{S}_x' + U \hat{S}_x'^2 + \widetilde{K}(\hat{S}_x' \hat{S}_y' + \hat{S}_y' \hat{S}_x'). \tag{9}$$

Now we can use the fact that the collective spin operators can be represented in terms of bosonic creation and annihilation operators as

$$\begin{aligned} \hat{S}_x' &= \frac{1}{2} \left(\hat{a}_2^\dagger \hat{a}_2 - \hat{a}_1^\dagger \hat{a}_1 \right), \\ \hat{S}_y' &= \frac{i}{2} \left(\hat{a}_2^\dagger \hat{a}_1 - \hat{a}_1^\dagger \hat{a}_2 \right) \\ \hat{S}_z' &= \frac{1}{2} \left(\hat{a}_2^\dagger \hat{a}_1 + \hat{a}_1^\dagger \hat{a}_2 \right). \end{aligned} \tag{10}$$

Using this mapping and the relation (3), it is then easy to see that the LMG Hamiltonian equals the Hamiltonian (2) with $L = 2$ oscillators up to a constant

$$\begin{aligned} H' = & \frac{\omega}{2} \left(\hat{a}_2^\dagger \hat{a}_2 - \hat{a}_1^\dagger \hat{a}_1 \right) + \frac{U}{2} \left(\hat{a}_2^{\dagger 2} \hat{a}_2^2 + \hat{a}_1^{\dagger 2} \hat{a}_1^2 \right) \\ & + \frac{\widetilde{K}}{4} \left[i(\hat{a}_2^\dagger \hat{a}_1 - \hat{a}_1^\dagger \hat{a}_2)(\hat{a}_2^\dagger \hat{a}_2 - \hat{a}_1^\dagger \hat{a}_1) + \text{h.c.} \right]. \end{aligned} \tag{11}$$

Supplementary Note 2

Synchronization for cold atoms in tilted optical lattices

We demonstrate how the quantum synchronization Hamiltonian arises for cold bosonic atoms in tilted optical lattices. The quantum dynamics of the atoms in the lattice potential is described by the second-quantized Hamiltonian

$$\hat{H} = \int \hat{\psi}^\dagger(x)\hat{H}_1\hat{\psi}(x)\,dx + \frac{U_0}{2}\int \hat{\psi}^\dagger(x)\hat{\psi}^\dagger(x)\hat{\psi}(x)\hat{\psi}(x)\,dx, \tag{12}$$

where $\hat{\psi}(x,t)$ is the bosonic field operator and we use the s-wave scattering approximation [6]. We consider a tilted or accelerated one-dimensional optical lattice, such that the single-particle Hamiltonian reads

$$\hat{H}_1 = -\frac{\hbar^2}{2m}\frac{\partial^2}{\partial x^2} + V(x) \tag{13}$$

with

$$V(x) = V_0\cos(2\pi x/d) + Fx, \tag{14}$$

where d is the lattice period. The one-dimensional limit can be realized by a tight confinement of the atoms in the radial directions, while the external field F can be realized by accelerating the entire lattice (see, e.g. [7]), by gravity or by magnetic gradient fields (see, e.g. [8, 9]). Alternatively, such a system is realized for the propagation of light beams in modulated photonic lattices, where the index of refraction is periodically modulated [10, 11].

The dynamics can be understood by expanding the field operators into the eigenstates of the single-particle Hamiltonian $\hat{H}_1$, the so-called Wannier-Stark resonance states [12, 13]. The linear system has a fundamental symmetry, it is invariant under a combined translation by the lattice period d and a shift of the energy by dF. The eigenstates are therefore organized in ladders,

$$\begin{aligned}\Psi_{\alpha,n}(x) &= \Psi_{\alpha,0}(x-dn),\\ E_{\alpha,n} &= E_{\alpha,0} + dFn,\end{aligned} \tag{15}$$

where Ψ is the wave function and E the energy of the eigenstates. Each ladder $\alpha = 0, 1, 2, \ldots$ roughly corresponds to one Bloch band in the field-free case and $n \in \mathbb{Z}$ labels the rung of the ladder. The fundamental time scale of the system is determined by the energy distance

of the rungs $E_{\alpha,n+1} - E_{\alpha,n} = dF$. In the non-interacting limit this gives rise to a periodic motion, the Bloch oscillations [7, 12–18] with the frequency $\omega_B = dF/\hbar$.

If the external field is not too strong, Landau-Zener tunneling between the bands can be neglected and the dynamics takes place in the ground ladder $\alpha = 0$ only [19]. Plugging the expansion of the field operator into the Wannier-Stark resonance states

$$\hat{\psi}(x) = \sum_{n=-\infty}^{+\infty} \hat{b}_n \Psi_{0,n}(x) \tag{16}$$

into equation (12) then yields the Hamiltonian

$$\hat{H} = \hbar\omega_B \sum_{n=-\infty}^{+\infty} n\, \hat{b}_n^\dagger \hat{b}_n + \frac{U_0}{2} \sum_{k\ell mn} \chi_{k,\ell;m,n} \hat{b}_k^\dagger \hat{b}_\ell^\dagger \hat{b}_m \hat{b}_n \tag{17}$$

with the overlap integrals

$$\chi_{k,\ell;m,n} = \int_{-\infty}^{+\infty} \Psi_{0,k}^*(x) \Psi_{0,\ell}^*(x) \Psi_{0,m}(x) \Psi_{0,n}(x)\, dx. \tag{18}$$

We note that this expansion is different from the one based on the Wannier functions which is commonly used in the analysis of the field-free case $F = 0$ [6]. Wannier functions are strongly localized in one well of the lattice such that nonlinear coupling terms between the different wells can be neglected. However, Wannier functions are no eigenstates such that there is a linear coupling term describing single-particle tunneling between adjacent wells. Wannier-Stark functions are much better suited for tilted or accelerated lattices: The periodicity of the single-particle dynamics becomes apparent and the effects of the nonlinearity can be analyzed rather directly [9, 20, 21].

The many-body-quantum dynamics described by the Hamiltonian (17) depends crucially on the overlap of the Wannier-Stark resonance states (18). A remarkable property of these states is their localization in real space, which increases with the lattice depth and the field strength. Therefore, several approximations can be made which greatly reduce the complexity of the interaction terms in the Hamiltonian (17). By definition, we have the permutational invariance

$$\begin{aligned} \chi_{k,\ell;m,n} &= \chi_{\ell,k;m,n} = \chi_{k,\ell;n,m}\,, \\ \chi_{k,\ell;m,n} &= \chi_{m,n;k,\ell}^*\,. \end{aligned} \tag{19}$$

Furthermore, we note that

$$\chi_{k,\ell;m,n} = \chi_{0,\ell-k;m-k,n-k} = \chi_{k-m,\ell-m;0,n-m} \tag{20}$$

due to the translational symmetry of the Wannier-Stark states. For a sufficiently deep lattice, only the on-site coupling and the coupling between nearest neighbor states are strong due to the strong localization of the Wannier-Stark states (cf. Supplementary Fig. **2** and [20]). Furthermore we find that

$$\mathrm{Im}(\chi_{n,n;n,n+1}),\ \mathrm{Im}(\chi_{n,n;n,n-1}) \approx 0,$$

$$\chi_{n,n;n,n+1} = \chi_{0,0;0,+1} \approx -\chi_{n,n;n,n-1} = -\chi_{0,0;0,-1}\,.$$

Using these approximation and neglecting all other interaction terms, the Hamiltonian (17) reduces to

$$\begin{aligned}\hat{H} = \hbar\omega_B \sum_{n=-\infty}^{+\infty} n\,\hat{b}_n^\dagger \hat{b}_n + \frac{U_0}{2}\chi_{0,0;0,0} \sum_{n=-\infty}^{+\infty} \hat{b}_n^\dagger \hat{b}_n^\dagger \hat{b}_n \hat{b}_n \\ +U_0\chi_{0,0;0,1} \sum_{n=-\infty}^{+\infty} \Big(\hat{b}_n^\dagger \hat{b}_n^\dagger \hat{b}_n \hat{b}_{n+1} - \hat{b}_n^\dagger \hat{b}_n^\dagger \hat{b}_n \hat{b}_{n-1} \\ + \hat{b}_n^\dagger \hat{b}_{n+1}^\dagger \hat{b}_n \hat{b}_n - \hat{b}_n^\dagger \hat{b}_{n-1}^\dagger \hat{b}_n \hat{b}_n\Big)\,. \end{aligned} \tag{21}$$

Shifting the phase of each mode by a constant factor,

$$\hat{b}_n = e^{in\pi/2}\hat{a}_n \tag{22}$$

we finally obtain the quantum synchronization Hamiltonian as discussed in our paper,

$$\begin{aligned}\hat{H} = \hbar\omega_B \sum_n n\,\hat{a}_n^\dagger \hat{a}_n + \frac{U}{2}\sum_n \hat{a}_n^\dagger \hat{a}_n^\dagger \hat{a}_n \hat{a}_n \\ +\frac{\widetilde{K}}{8}\sum_n \Big[i(\hat{a}_{n+1}^\dagger \hat{a}_n^\dagger - \hat{a}_n^\dagger \hat{a}_{n+1})(\hat{a}_{n+1}^\dagger \hat{a}_{n+1} - \hat{a}_n^\dagger \hat{a}_n) + \mathrm{h.c.}\Big]\end{aligned} \tag{23}$$

with $U = U_0 \times \chi_{0,0;0,0}$ and $\widetilde{K} = 4U_0 \times \chi_{0,0;0,1}$. We note that the phase shift (22) has no implications for the physics described by the model. It has been introduced only for the sake of notational convenience, to allow for a better comparability with the established Kuramoto model.

In the paper we consider the dynamics of an initially pure Bose-Einstein condensate, i.e. a product state where all bosonic atoms are in the same single-partice quantum state

$$\begin{aligned}|\mathrm{BEC}\rangle &= \frac{1}{\sqrt{N!}}\left(\sum_{j=1}^{L} c_j \hat{b}_j^\dagger\right)^N |0\rangle. \\ &= \frac{1}{\sqrt{N!}}\left(\sum_{j=1}^{L} e^{-ij\pi/2} c_j \hat{a}_j^\dagger\right)^N |0\rangle.\end{aligned} \tag{24}$$

In particular, we consider a condensate which is initally at rest, i.e. has homogeneous phases, and is widely distributed in space such that $c_j = 1/\sqrt{L}$ for $j = 1, \ldots, L$.

In experiments with ultracold atoms one commonly measures the momentum space density $\rho(k,t) = \langle \hat{\psi}^\dagger(k)\hat{\psi}(k)\rangle$ using the time-of-flight technique. Using the expansion (16) and the translational symmetry of the Wannier-Stark states

$$\begin{aligned} &\Psi_{\alpha,n}(x) = \Psi_{\alpha,0}(x - dn) \\ \Rightarrow \quad &\Psi_{\alpha,n}(k) = e^{-inkd}\Psi_{\alpha,0}(k) \end{aligned} \tag{25}$$

the momentum density reads

$$\begin{aligned} \rho(k,t) &= |\Psi_{0,0}(k)|^2 \sum_{n,\ell} e^{-i(n-\ell)kd} \langle \hat{b}_\ell^\dagger \hat{b}_n \rangle_t \\ &= |\Psi_{0,0}(k)|^2 \sum_{n,\ell} e^{-i(n-\ell)kd} e^{i(n-\ell)\pi/2} \langle \hat{a}_\ell^\dagger \hat{a}_n \rangle_t \,. \end{aligned} \tag{26}$$

The long-time evolution of the quantum many-body system is thus determined by the coherences $\langle \hat{a}_\ell^\dagger \hat{a}_n \rangle_t$. As shown in our paper, synchronization can induce long-time coherence. To understand how this manifests in laboratory experiments, we briefly review the dynamics for $K = 0$ in the following.

In the linear case $U = K = 0$ the coherences evolve as

$$\langle \hat{b}_\ell^\dagger \hat{b}_n \rangle_t = e^{-i(n-\ell)\omega_B t} \langle \hat{b}_\ell^\dagger \hat{b}_n \rangle_0 \tag{27}$$

which can be directly seen from integrating the Heisenberg equation,

$$\frac{d}{dt}\hat{b}_n = \frac{i}{\hbar}[\hat{H}, \hat{b}_n] = -i\omega_B n\, \hat{b}_n. \tag{28}$$

Hence, the momentum space density of the atoms is given by

$$\rho(k,t) = |\Psi_{0,0}(k)|^2 \sum_{n,\ell} e^{-i(n-\ell)(kd-\omega_B t)} \langle \hat{b}_\ell^\dagger \hat{b}_n \rangle_0. \tag{29}$$

In this formulation one can directly see that the quantum dynamics is fully periodic, which is commonly referred to as Bloch oscillations [7, 12–18]. If t is an integer multiple of the Bloch time $T_B = 2\pi/\omega_b$, we have $e^{-i(n-\ell)\omega_B t} = 1$ such that

$$\rho(k, t = mT_B) = \rho(k, 0) \qquad \forall m \in \mathbb{Z}. \tag{30}$$

For an initially pure BEC (24), the coherences of this state are simply given by $\langle \hat{b}_\ell^\dagger \hat{b}_n \rangle_0 = N c_\ell^* c_n$ such that the momentum density (29) can be further simplified to

$$\rho(k,t) = |\Psi_{0,0}(k)|^2 \times |\widetilde{C}(kd + \omega_B t)|^2, \tag{31}$$

where $\widetilde{C}$ denotes the discrete Fourier transform of the amplitudes c_n:

$$\widetilde{C}(y) = \sum_{j=1}^{L} c_j e^{-ijy}. \tag{32}$$

If the BEC is initially distributed widely distributed in space, then the Fourier transform $|\widetilde{C}|^2$ is a 'comb' function, i.e. a periodic arrangement of sharp peaks. Hence we obtain a particular simple picture of Bloch oscillations in momentum space: A comb function is moving under the envelope given by $|\Psi_{0,0}(k)|^2$ (cf. Supplementary Fig. **3**).

In the regime of strong on-site interaction U and no coupling $K = 0$, all non-diagonal coherences tend to zero, $\langle \hat{b}_\ell^\dagger \hat{b}_n \rangle \to 0$ for $\ell \neq n$ [20, 22–24], and the momentum density reads

$$\rho(k,t) \to |\Psi_{0,0}(k)|^2 \sum_{n=1}^{L} \langle \hat{b}_n^\dagger \hat{b}_n \rangle = N |\Psi_{0,0}(k)|^2. \tag{33}$$

Revivals are possible in finite systems for longer time scales. The Wannier-Stark resonance state $\Psi_{0,0}(k)$ roughly extends over the first Brillouin zone $k \in [-\pi/d, +\pi/d]$ (cf. Supplementary Fig. **3**). Strong on-site interactions thus lead to a delocalization of $\rho(k,t)$ over the entire first Brillouin zone. A localization in momentum space thus indicates the presence of persistent *quantum* correlations, which emerge due to *classical* synchronization, as discussed in the paper.

Supplementary Note 3

The mean-field limit

The mean-field equations of motion for the amplitudes $c_\ell = \langle \hat{a}_\ell \rangle$ are most easily derived from the Heisenberg equation

$$\frac{d}{dt}\hat{a}_n = \frac{i}{\hbar}[\hat{H}, \hat{a}_n] \tag{34}$$

with the Hamiltonian

$$\hat{H} = \sum_{j=1}^{L} \omega_j \hat{a}_j^\dagger \hat{a}_j + \frac{U}{2}\hat{a}_j^{\dagger 2}\hat{a}_j^2 + \hat{H}_{\rm s}\,, \tag{35}$$

$$\hat{H}_{\rm s} = \sum_{j,\ell=1}^{L} \frac{\widetilde{K}_{j,\ell}}{8}\left[i(\hat{a}_j^\dagger \hat{a}_\ell - \hat{a}_\ell^\dagger \hat{a}_j)(\hat{a}_j^\dagger \hat{a}_j - \hat{a}_\ell^\dagger \hat{a}_\ell) + \text{h.c.}\right].$$

Throughout this paper we use scaled units with $\hbar = 1$, thus measuring energy in units of frequency. The relevant commutators can be evaluated in a straightforward way with the result

$$[\hat{a}_n, \hat{a}_j^\dagger \hat{a}_j] = \delta_{n,j}\hat{a}_n \tag{36}$$

$$[\hat{a}_n, \hat{a}_j^{\dagger 2}\hat{a}_j^2] = 2\delta_{n,j}\hat{a}_n^\dagger \hat{a}_n^2 \tag{37}$$

$$[\hat{a}_n, \hat{a}_j^\dagger \hat{a}_\ell \hat{a}_j^\dagger \hat{a}_j] = 2\delta_{n,j}\hat{a}_n^\dagger \hat{a}_n \hat{a}_j \tag{38}$$

$$[\hat{a}_n, \hat{a}_j^\dagger \hat{a}_\ell \hat{a}_\ell^\dagger \hat{a}_\ell] = (1-\delta_{n,j})\hat{a}_\ell^\dagger \hat{a}_\ell^2 + (1-\delta_{n,\ell})\hat{a}_j^\dagger \hat{a}_\ell^2 + \delta_{n,j}\hat{a}_\ell \tag{39}$$

such that we obtain

$$i\frac{d}{dt}\hat{a}_n = \omega_n \hat{a}_n + U\hat{a}_n^\dagger \hat{a}_n^2 + \sum_{j=1}^{L}\frac{\widetilde{K}_{nj}}{2i}(\hat{a}_j^\dagger \hat{a}_n^2 + \hat{a}_j^\dagger \hat{a}_j^2 - 2\hat{a}_n^\dagger \hat{a}_n \hat{a}_j). \tag{40}$$

Taking the expectation value on both sides of the equation then yields

$$\begin{aligned} i\frac{d}{dt}\langle \hat{a}_n \rangle = {} & \omega_n \langle \hat{a}_n \rangle + U\langle \hat{a}_n^\dagger \hat{a}_n^2 \rangle \\ & + \sum_{j=1}^{L}\frac{\widetilde{K}_{nj}}{2i}(\langle \hat{a}_j^\dagger \hat{a}_n^2 \rangle + \langle \hat{a}_j^\dagger \hat{a}_j^2 \rangle - 2\langle \hat{a}_n^\dagger \hat{a}_n \hat{a}_j \rangle) \end{aligned} \tag{41}$$

This equation includes the three point functions of the form $\langle \hat{a}_j^\dagger \hat{a}_k \hat{a}_\ell \rangle$ on the right-hand side. This is a general feature of quantum many-body system: The Heisenberg equations induce an infinite hierarchy of coupled equations for the expectation values. To obtain a closed set

of equations we must truncate this hierarchy at some point by approximating the three-point functions in terms of one-point functions

$$\langle \hat{a}_j^\dagger \hat{a}_k \hat{a}_\ell \rangle \approx \langle \hat{a}_j^\dagger \rangle \langle \hat{a}_k \rangle \langle \hat{a}_\ell \rangle = c_j^* c_k c_\ell, \tag{42}$$

where the asterisk denotes complex conjugation. The truncation is generally valid for Bose-Einstein condensates with a high number of particles, as the error vanishes as $1/N$. A discussion of the validity of this truncation and other approaches can be found in [25] and the references therein. We finally arrive at the mean-field equations of motion,

$$i\frac{dc_n}{dt} = \omega_n c_n + U|c_n|^2 c_n + \sum_{j=1}^{L} \frac{\widetilde{K}_{nj}}{2i} (c_j^* c_n^2 + |c_j|^2 c_j - 2|c_n|^2 c_j). \tag{43}$$

For further analysis we decompose the complex numbers c_n into amplitude and phase

$$c_n = |c_n| e^{-i\phi_n}, \tag{44}$$

that is,

$$|c_n| = (c_n^* c_n)^{1/2} \quad \text{and} \quad \phi_n = -\arctan\left(\frac{\mathrm{Im}(c_n)}{\mathrm{Re}(c_n)}\right). \tag{45}$$

Within the mean-field approximation, the squared modulus $|c_n|^2$ corresponds to the number of excitations or atoms per mode, respectively:

$$|c_n|^2 = \langle \hat{a}_n^\dagger \rangle \langle \hat{a}_n \rangle \approx \langle \hat{a}_n^\dagger \hat{a}_n \rangle. \tag{46}$$

The equations of motion for the amplitudes and phases are then derived from equation (43), which leads to

$$\frac{d}{dt}|c_n|^2 = -\sum_{j=1}^{L} \widetilde{K}_{nj} (|c_j|^2 - |c_n|^2) |c_j||c_n| \cos(\phi_n - \phi_j) \tag{47}$$

$$\frac{d}{dt}\phi_n = \omega_n + U|c_n|^2 + \sum_{j=1}^{L} \frac{\widetilde{K}_{nj}}{2} \frac{|c_j|}{|c_n|} (3|c_n|^2 - |c_j|^2) \sin(\phi_j - \phi_n). \tag{48}$$

The first line shows that the amplitudes remains constant if all have the same value

$$\frac{d}{dt}|c_n|^2 \equiv 0 \quad \text{if} \quad |c_j|^2 = |c_n|^2 \; \forall\, j, n = 1, \ldots, L. \tag{49}$$

Denoting the total number of excitations as

$$N = \sum_{\ell=1}^{L} |c_\ell|^2 \tag{50}$$

we thus find that the toric manifolds

$$T_N = \left\{ \mathbf{c} \in \mathbb{C}^L \middle| \, |c_\ell|^2 = N/L \, \forall \ell = 1 \ldots, L \right\} \tag{51}$$

are invariant under the flow generated by the equations (43). On a torus with a given number of excitations N only the phases evolve according to equation

$$\frac{d\phi_n}{dt} = \omega_n + U\frac{N}{L} + \sum_{j=1}^{L} K_{nj} \sin(\phi_j - \phi_n), \tag{52}$$

where $K_{nj} = N\widetilde{K}_{nj}/L$. This exactly constitutes the celebrated Kuramoto model [26–28].

Supplementary Note 4

Beyond mean-field

The dynamics of quantum fluctuations beyond mean-field and the depletion of the condensate mode can be analyzed in a Bogoliubov-de Gennes approach. To this end we decompose the bosonic annihilation operators into the condensate mode c_n and the remaining quantum fluctuations, described by the operator $\hat{b}_n$. Together, this gives the ansatz:

$$\hat{a}_n = c_n + \hat{b}_n \,. \tag{53}$$

Assuming an almost pure condensate with N atoms, c_n is of order $\sqrt{N}$ while the fluctuations $\hat{b}_n$ are of order 1. In the mean-field limit one furthermore assumes that $N \to \infty$, while the macroscopic interaction strength $UN/L = g$ and $N\tilde{K}_{j\ell}/L = K_{j\ell}$ tend to a constant value. We can thus insert the ansatz (53) into the Heisenberg equations of motion (41) and sort the equation according to the order of the atom number N. To leading order $N^{1/2}$ one recovers the mean-field equations of motion (43). In next to leading order one obtains the Bogoliubov-de Gennes equations

$$i\frac{d}{dt}\begin{pmatrix}\hat{b}_n\\ \hat{b}_n^\dagger\end{pmatrix} = \sum_{j=1}^{L} \underbrace{\begin{pmatrix}\zeta_{nj} & \eta_{nj}\\ -\eta_{nj}^* & -\zeta_{nj}^*\end{pmatrix}}_{=:\mathcal{L}_{nj}} \begin{pmatrix}\hat{b}_j\\ \hat{b}_j^\dagger\end{pmatrix} \tag{54}$$

with the matrix elements

$$\zeta_{nj} = -i\widetilde{K}_{nj}(|c_j|^2 - |c_n|^2) + \delta_{nj}\left[\omega_n + 2U|c_n|^2 - i\sum_{\ell=1}^{L}\widetilde{K}_{n\ell}(c_\ell^* c_n - c_n^* c_\ell)\right] \tag{55}$$

$$\eta_{nj} = -\frac{i}{2}\widetilde{K}_{nj}(c_j^2 + c_n^2) + \delta_{nj}\left[U c_n^2 + i\sum_{\ell=1}^{L}\widetilde{K}_{n\ell}c_n c_\ell\right]. \tag{56}$$

On the Kuramoto manifold we have $c_n = \sqrt{N/L}e^{-i\phi_n}$ and the matrix elements simplify to

$$\begin{aligned}
\zeta_{nj} &= \delta_{nj}\left[\omega_n + 2\frac{UN}{L} + 2\sum_{\ell} K_{n\ell}\sin(\phi_\ell - \phi_n)\right] \\
\eta_{nj} &= -\frac{i}{2}K_{nj}\left(e^{-2i\phi_n} + e^{-2i\phi_j}\right) + \delta_{nj}\left[\frac{UN}{L}e^{-2i\phi_n} + i\sum_{\ell} K_{n\ell}e^{-i(\phi_n+\phi_\ell)}\right].
\end{aligned} \tag{57}$$

Now the main question is whether this equation is dynamically stable or not, i.e. whether the quantum fluctuations remain bounded or grow exponentially. If the Bogoliubov-de Gennes operator

$$\mathcal{L} = \begin{pmatrix} \mathcal{L}_{11} & \mathcal{L}_{12} & \dots \\ \mathcal{L}_{21} & \mathcal{L}_{22} & \dots \\ \vdots & \vdots & \ddots \end{pmatrix} \tag{58}$$

is time-independent, this can be directly read of from its eigenvalues $\lambda_{1,\dots,2N}$. If all eigenvalues are real, then the condensate is stable and the number of non-condensed atoms can grow only slowly. If an eigenvalue becomes complex with $\mathrm{Im}(\lambda_n) > 0$ then the Bogoliubov-de Gennes equations predict an exponential growth of the associated eigenmode. The condensate is dynamically unstable and number fluctuations typically become very large. If $\mathcal{L}$ is explicitly time-dependent, more care has to be taken. The Bogoliubov-de Gennes equation (54) can be formally solved as

$$\begin{pmatrix} \hat{b}_1(t) \\ \vdots \end{pmatrix} = \hat{\mathcal{T}} e^{-i\int_0^t \mathcal{L}(t')dt'} \begin{pmatrix} \hat{b}_1(0) \\ \vdots \end{pmatrix}, \tag{59}$$

where $\hat{\mathcal{T}}$ denotes time ordering. We are particularly interested in the case where the mean-field dynamics is periodic with period T. Then we can define a time-averaged Bogoliubov-de Gennes operator $\bar{\mathcal{L}}$ as

$$e^{-i\bar{\mathcal{L}}T} = \hat{\mathcal{T}} e^{-i\int_0^T \mathcal{L}(t')dt'} \tag{60}$$

Using this definition we obtain the same condition for dynamical instability as in the time-independent case: The condensate is stable if all eigenvalues of $\bar{\mathcal{L}}$ are real, it becomes unstable if an eigenvalue becomes complex with $\mathrm{Im}(\lambda_n) > 0$. We note that in this case the real parts of the eigenvalues are defined only modulo $2\pi/T$.

The Kuramoto model was originally introduced and solved for the case of many globally connected oscillators [26]. In this case we can analytically estimate the eigenvalues of the Bogoliubov-de Gennes operator and thus derive criteria for the growth of quantum fluctuations in the quantum Kuramoto model. So we consider the globally connected case $K_{nj} = K/L$ on the Kuramoto manifold in the limit of many coupled oscillators $L \to \infty$ with a fixed density $\nu = N/L$ and define the Kuramoto phase order parameter

$$re^{i\gamma} = \frac{1}{L}\sum_{\ell=1}^{L} e^{i\phi_\ell}\,. \tag{61}$$

In the generic case the oscillators relax to a steady state with a fixed value of r. The global phase γ is also constant in time if we transform to an appropriate co-rotating frame of reference,

$$
\begin{aligned}
c_n &\to c_n e^{-i\Omega t} \qquad \Rightarrow \qquad \phi_n \to \phi_n + \Omega t \\
\hat{b}_n &\to \hat{b}_n e^{-i\Omega t}
\end{aligned}
\tag{62}
$$

where Ω is given by the mean frequency of the oscillators,

$$
\Omega = \frac{1}{L}\sum_{\ell=1}^{N} \omega_\ell + U\nu \; = \bar{\omega} + U\nu. \tag{63}
$$

The mean-field equations of motion on the Kuramoto manifold the read

$$
\frac{d\phi_n}{dt} = \omega_n + U\nu - \Omega + Kr\sin(\gamma - \phi_n), \tag{64}
$$

and the coefficients of the Bogoliubov-de Gennes operator (56) can be simplified to

$$
\begin{aligned}
\zeta_{nj} &= \delta_{nj}\left(\omega_n - \Omega + 2U\nu + 2Kr\sin(\gamma - \phi_n)\right) \\
\eta_{nj} &= \delta_{nj}\left(U\nu e^{-2i\phi_n} + iKre^{-i(\phi_n+\gamma)}\right) - \frac{iK}{2L}\left(e^{-2i\phi_n} + e^{-2i\phi_j}\right).
\end{aligned}
\tag{65}
$$

We observe that in the limit $L \to \infty$ all off-diagonal elements of η vanish as L^{-1} such that both ζ and η become diagonal. Thus the the Bogoliubov-de Gennes equation for the individual oscillators decouple,

$$
i\frac{d}{dt}\begin{pmatrix} \hat{b}_n \\ \hat{b}_n^\dagger \end{pmatrix} = \mathcal{L}_{nn}\begin{pmatrix} \hat{b}_n \\ \hat{b}_n^\dagger \end{pmatrix}, \tag{66}
$$

and we can analyze the stability of all oscillators separately. To evaluate the stability, we have to distinguish between phase-locked and drifting oscillators. All oscillators with $|\omega_n + U\nu - \Omega| \le Kr$ are phase-locked, i.e. the phase ϕ_n assumes a fixed value given by the equation

$$
\omega_n + U\nu - \Omega + Kr\sin(\gamma - \phi_n) = 0. \tag{67}
$$

Thus the Bogoliubov-de Gennes operator $\mathcal{L}_n$ is time-independent with

$$
\begin{aligned}
|\zeta_{nn}|^2 &= (\omega_n - \Omega)^2 \\
|\eta_{nn}|^2 &= (Kr)^2 - (U\nu)^2 - 2U\nu(\omega_n - \Omega)
\end{aligned}
$$

such that we can readily evaluate its eigenvalues with the result

$$\lambda_{n,\pm} = \pm\sqrt{|\zeta_{nn}|^2 - |\eta_{nn}|^2} \tag{68}$$

$$= \pm\sqrt{(\omega_n + U\nu - \Omega)^2 - (Kr)^2}\,. \tag{69}$$

For all phase-locked oscillators with $|\omega_n + U\nu - \Omega| < Kr$ this expression is purely imaginary, such that quantum fluctuations grow. The growth rate tends to zero, $\mathrm{Im}(\lambda_{n,\pm}) \to 0$, on the border of the phase-locking region at $|\omega_n + U\nu - \Omega| = Kr$.

Oscillators with $|\omega_n + U\nu - \Omega| > Kr$ do not phase-lock but 'drift' around the unit cycle. The mean-field dynamics is periodic such that we have to analyze the eigenvalues of the averaged operator $\mathcal{L}_{nn}$ as discussed above. In this case the time-ordered integral in equation (60) is evaluated numerically to obtain the eigenvalues $\lambda_{n,\pm}$. We observe a non-zero growth rate in the immediate vicinity of the phase-locking region while $\lambda_{n,\pm}$ becomes purely real for all other drifting oscillators (cf. Supplementary Fig. **4**).

We stress that a dynamical instability is possible only if the phase order parameter r does not vanish. For $r = 0$ all oscillators are drifting according to $\phi_n(t) = (\omega_n + U\nu - \Omega)t$ and the Bogoliubov-de Gennes operator reads

$$\mathcal{L}_{nn}(t) = \begin{pmatrix} \omega_n - \Omega + 2U\nu & g e^{-2i(\omega_n + U\nu - \Omega)t} \\ -U\nu e^{+2i(\omega_n + U\nu - \Omega)t} & -\omega_n + \Omega - 2U\nu \end{pmatrix}. \tag{70}$$

The off-diagonal contributions average out such that the eigenvalues of $\bar{\mathcal{L}}_{nn}$ are purely real.

Supplementary References

[1] Lipkin, H. J., Meshkov, N. and Glick, A. J. Validity of many-body approximation methods for a solvable model: (I). Exact solutions and perturbation theory. *Nuclear Physics* **62**, 188–198 (1965).

[2] Dusuel, S. and Vidal, J. Finite-size scaling exponents of the Lipkin-Meshkov-Glick model. *Phys. Rev. Lett.* **93**, 237204 (2004).

[3] Unanyan, R. G. and Fleischhauer, M. Decoherence-free generation of many-particle entanglement by adiabatic ground-state transitions. *Phys. Rev. Lett.* **90**, 133601 (2003).

[4] Chen, G., Liang, J. Q. and Jia, S. Interaction-induced Lipkin-Meshkov-Glick model in a Bose-Einstein condensate inside an optical cavity. *Opt. Express* **17**, 19682–19690 (2009).

[5] Larson, J. Circuit QED scheme for the realization of the Lipkin-Meshkov-Glick model. *Europhys. Lett. (Europhysics Letters)* **90**, 54001 (2010).

[6] Jaksch, D. and Zoller, P. The cold atom Hubbard toolbox. *Annals of Physics* **315**, 52–79 (2005).

[7] Dahan, M. B., Peik, E., Reichel, J., Castin, Y. and Salomon, C. Bloch oscillations of atoms in an optical potential. *Phys. Rev. Lett.* **76**, 4508 (1996).

[8] Gustavsson, M. *et al.* Control of interaction-induced dephasing of Bloch oscillations. *Phys. Rev. Lett.* **100**, 080404 (2008).

[9] Gustavsson, M. *et al.* Interference of interacting matter waves. *New J. Phys.* **12**, 065029 (2010).

[10] Bromberg, Y., Lahini, Y., Morandotti, R. and Silberberg, Y. Quantum and classical correlations in waveguide lattices. *Phys. Rev. Lett.* **102**, 253904 (2009).

[11] Garanovich, I. L., Longhi, S., Sukhorukov, A. A. and Kivshar, Y. S. Light propagation and localization in modulated photonic lattices and waveguides. *Physics Reports* **518**, 1 – 79 (2012).

[12] Glück, M., Kolovsky, A. R. and Korsch, H. J. Wannier-Stark resonances in optical and semiconductor superlattices. *Physics Reports* **366**, 103–182 (2002).

[13] Hartmann, T., Keck, F., Korsch, H. J. and Mossmann, S. Dynamics of Bloch oscillations. *New J. Phys.* **6**, 2 (2004).

[14] Feldmann, J. *et al.* Optical investigation of Bloch oscillations in a semiconductor superlattice.

Phys. Rev. B **46**, 7252 (1992).

[15] Wilkinson, S. R., Bharucha, C. F., Madison, K. W., Niu, Q. and Raizen, M. G. Observation of atomic Wannier-Stark ladders in an accelerating optical potential. *Phys. Rev. Lett.* **76**, 4512 (1996).

[16] Morandotti, R., Peschel, U., Aitchinson, J. S., Eisenberg, H. S. and Silberberg, Y. Experimental observation of linear and nonlinear optical Bloch oscillations. *Phys. Rev. Lett.* **83**, 4756 (1999).

[17] Pertsch, T., Dannberg, P., Elflein, W., Bräuer, A. and Lederer, F. Optical Bloch oscillations in temperature tuned waveguide arrays. *Phys. Rev. Lett.* **83**, 4752 (1999).

[18] Christodoulides, D. N., Lederer, F. and Silberberg, Y. Discretizing light behaviour in linear and nonlinear waveguide lattices. *Nature* **424**, 817–823 (2003).

[19] Parra-Murillo, C. A., Madroñero, J. and Wimberger, S. Two-band Bose-Hubbard model for many-body resonant tunneling in the Wannier-Stark system. *Phys. Rev. A* **88**, 032119 (2013).

[20] Thommen, Q., Garreau, J. C. and Zehnlé, V. Classical chaos with Bose-Einstein condensates in tilted optical lattices. *Phys. Rev. Lett.* **91**, 210405 (2003).

[21] Witthaut, D., Werder, M., Mossmann, S. and Korsch, H. J. Bloch-oscillations of Bose-Einstein condensates: Breakdown and revival. *Phys. Rev. E* **71**, 036625 (2005).

[22] Buchleitner, A. and Kolovsky, A. R. Interaction-induced decoherence of atomic Bloch oscillations. *Phys. Rev. Lett.* **91**, 253002 (2003).

[23] Kolovsky, A. R. New Bloch period for interacting cold atoms in 1D optical lattices. *Phys. Rev. Lett.* **90**, 213002 (2003).

[24] Meinert, F. *et al.* Interaction-induced quantum phase revivals and evidence for the transition to the quantum chaotic regime in 1D atomic Bloch oscillations. *Phys. Rev. Lett.* **112**, 193003 (2014).

[25] Tikhonenkov, I., Anglin, J. and Vardi, A. Quantum dynamics of Bose-Hubbard hamiltonians beyond the Hartree-Fock-Bogoliubov approximation: The Bogoliubov back-reaction approximation. *Physical Review A* **75**, 013613 (2007).

[26] Kuramoto, Y. Self-entrainment of a population of coupled non-linear oscillators. In Araki, H. (ed.) *International Symposium on on Mathematical Problems in Theoretical Physics*, Lecture Notes in Physics Vol. 39, 420 (Springer, New York, 1975).

[27] Strogatz, S. H. From Kuramoto to Crawford: Exploring the onset of synchronization in

populations of coupled oscillators. *Physica D: Nonlinear Phenomena* **143**, 1–20 (2000).

[28] Acebrón, J. A., Bonilla, L. L., Pérez Vicente, C. J., Ritort, F. and Spigler, R. The Kuramoto model: A simple paradigm for synchronization phenomena. *Rev. Mod. Phys.* **77**, 137–185 (2005).
