# Supplementary material for: Classical synchronization indicates persistent entanglement in isolated quantum systems
Source: Nat Commun. 2017 Apr 12;8:14829. doi: 10.1038/ncomms14829 (PMC5394286; doi:10.1038/ncomms14829)
Supplement: Peer Review File [file ncomms14829-s2.pdf]

Reviewer #1 (Remarks to the Author):

This manuscript reports the analysis of a many-body system to show the connection between the emergence of synchronization and of entanglement.
The Hamiltonian (1) is considered, mainly in the case of 2 sites, to show the presence of entanglement and phase-locking above a critical value of the non-linear coupling (Figures 1 and 2). The corresponding classical system displays constant site amplitudes, with phase difference governed by the Kuramoto model. The model for N sites and global constant couplings is also shown to synchronize above a critical coupling (Fig.3). Quantum fluctuations (for global coupling) grow at a maximum rate given by the control parameter r (Eq.5). For local near neighbors couplings, this model can represent a BEC in a tilted potential and some results for L=4 sites are shown in Fig.4, displaying phase-locking and entanglement as witnessed by squeezed variance in the numbers difference.

The work is sound and the subject interesting. There is some fragmentation in presenting general conclusions: indeed some of them are for N=2, some for large systems (L), some for BEC (with near neighbors couplings) and some others for all-to-all couplings... Nevertheless all results are clearly exposed in the
main manuscript and several details are added in the Supplementary Information file.

To the best of my knowledge synchronization has not been considered in this specific model. On the other hand, there have been several works on quantum synchronization and the authors do not properly refer to the state of the art on the subject.

The main advance of this work, as claimed by the authors, would be to show the "first" connection between synchronization and entanglement. This, however, is not a novelty and the authors should properly refer to previous works (not more specific than the particular case analyzed in this manuscript) where this question was already discussed. Reference [27], that is actually cited, also discusses the connection between synchronization and
entanglement, showing that actually they do not always coincide. Furthermore, a relation between synchronization and quantum correlations or entanglement, is shown for instance, in Manzano et al, Sci. Rep. 3, 1439 (2013), Lee and Cross, PRA 88, 013834 (2013), Giorgi et al, PRA 88, 042115 (2013), Lee et al, PRE 89, 022913 (2014).... The results of this manuscript refer to a
specific model where the threshold for entanglement and for synchronization coincide. Being a connection between these features already reported in some previous works, the authors should better identify the novelty and importance of their work with respect to the others.

Looking at other results of this work, the identification of a Kuramoto model for the dynamics of a quantum system in the classical limit is also not new. Indeed, both the mapping into Kuramoto models and its absence have been reported in the literature: see, for instance, the case of of opto-mechanical
systems Heinrich et al. PRL 107, 043603 (2011), and Holmes et al., PRE 5, 066203 (2012), respectively.

Finally, the authors claim at page 9 that the absence of dissipation in this model, with respect to few other references they cite, represents an important advance. As a matter of fact, I do not find this to be really a key point. In general, non-linear dynamical systems with reciprocal coupling are susceptible to synchronize, and dissipation may be important or not in creating self-sustained oscillations. Synchronization can be studied in absence of dissipation, even in quantum systems, see for instance Liu et al., EPL, 103, 17007 (2013).
Furthermore, for the question addressed here by the authors, the absence of dissipation (and therefore of decoherence) and presence of non-linear couplings would naturally favors the presence of entanglement. So, I would actually find more interesting (or surprising) a connection between synchronization and entanglement in presence (and in spite) of dissipation.

Finally, in my opinion this manuscript is technically sound but I do not find it represents a substantial advance on this topics and I am not recommending its publication in Nature Communications.

Reviewer #2 (Remarks to the Author):

This is a paper that studies a model quantum many-body system as described by the Hamiltonian presented in Equation 1 of the manuscript. The model contains a set of coupled boson oscillators, with intrinsic frequency, onsite (self) interactions, and a specific form of oscillator-oscillator coupling. It should be emphasized that the problem studied is that of an isolated quantum system (i.e. the dynamics that is considered is that for a system that does not contain dissipation).

Firstly, let me comment on the topic. The main point is to make a connection between the extensively studied synchronization in networks of classical oscillators, with the problem of synchronization in quantum many-body physics. The later is much less explored and is difficult to treat exactly, due in part to the exponential dimensionalilty of the Hilbert space and the development of quantum entanglement and quantum correlations. This paper makes a strong argument for the link between the synchronization transition in the analogous classical system, and the development of entanglement and squeezing in the quantum dynamics.

Although this is certainly not the first time this connection has been explored, I believe the paper provides a significant contribution and focuses on an elegant system where robust connections can be made. The simplicity arises from the fact that the mean-field limit of the quantum system can be identified with a system of Kuramoto oscillators, which is the standard framework for approaching a description of classical synchronization. Furthermore, in the limit of two oscillators, the authors show that the quantum system reduces to a standard Lipkin-Meshkov-Glick model that may be familiar to some. I think it is particularly interesting to see the calculated behavior of quantum fluctuations around the critical phase locking points.

The paper is very well written, and I found it clear to follow, both the story and the mathematical details. The authors have paid considerable attention to the presentation.

My main concern with this paper is really a fundamental one, and perhaps this is not a topic of criticism of the manuscript per se, but is important at a fundamental level. The absence of dissipation in this system means that the evolution is reversible Hamiltonian dynamics, and therefore the irreversible quantum synchronization that appears in the quantum dynamics of open quantum systems (for example lasers) is fundamentally different. It is known that the presence of dissipation rapidly leads to attenuation of entanglement and quantum correlations, so I wonder to what extent the conclusions of this paper rely on the fact that the system considered is completely isolated. This may affect the potential application of the results to a diverse set of physical systems that people consider.

My own personal preference is that hbar should not be set to one, as is done here, and that time and energy for example should have their usual dimensions.

The figures are reasonably clear, and a thorough list of references to previous work is provided. The authors consider a physical system in which the model could be implemented as a Bose quantum gas in a tilted optical lattice, looking at momentum space localization. I think this helps to make the discussion less abstract, and potentially could be of interest to AMO experimenters.

Overall, I think this is a well-written paper, addressing an interesting topic that is receiving considerable interest from a broad field of physicists who have backgrounds in traditionally distinct

areas. I recommend it for publication.

Reviewer #3 (Remarks to the Author):

In their manuscript "Quantum signatures of synchronization" the authors study the dynamics of a many-bodied quantum Schrodinger equations. Remarkably, they draw a clear link between the quantum properties associated with the aforementioned system, including entanglement, coherence, squeezing, etc., and the synchronization dynamics of classical synchronization, specifically the Kumamoto model. In particular, they derive the equations of motion of the Kumamoto model directly from the Schrodinger equation and show that, for instance, entanglement in the quantum system emerges precisely at the onset of synchronization in the classical system. In addition to these theoretical results, the authors suggest experiments where their results may be observed (detailing the theoretical backing in the SM).

The manuscript is well-crafted, the analysis is significant, and most importantly the research is novel. Given that this work represents the first (to my knowledge) such direct link between classical synchronization and quantum dynamics, I recommend publication in Nature Communications.

A few small points:

One of the most central links between the quantum and classical systems (i.e., Eqs. (1) and (3)) is the frequency terms \omega_l. Can the authors give a brief interpretation of their significance in Eq. (1) and the physical translation to Eq. (3). Moreover, in the example in the manuscript the authors use Lorentzian distributed frequencies. Is this choice made for a specific reason? Does the Lorentzian itself have a physical meaning?

From Eq. (3) to Eq. (6) the authors change the subscripts from l to n. Is there a reason for this. (I believe that my initial confusion came from assuming that l = 1,...,L and n = 1,...,N.)

A small correction: below Eq. (6) the authors state that phase-locking occurs when |\omega_n-UN/L| \le Kr, however this is not true if the mean <\omega_n-UN/L> is not zero. If the frequencies themselves are initially set to have mean zero, then since UN/L is a uniform shift for all oscillators then the criteria is just |\omega_n| \le Kr.

In the case of the Bose-Einstein optical lattices the energies \omega_n, and thus the frequencies, are uniformly distributed, \omega_n=\omega_B*n. I am curious about the phase-transition to synchronization in the large system limit, where the distribution would be uniform. In the paper referred below it is shown that the phase-transition will be first-order. Does this have any physical significance?

D. Pazo, "Thermodynamic limit of the first-order phase transition in the Kuramoto model," Phys. Rev. E 72, 046211 (2005).

<u>Replies to the reviewers' comments on manuscript NCOMMS-16-11385</u>

by Dirk Witthaut, Sandro Wimberger, Raffaela Burioni and Marc Timme

<u>Replies to the comments of all reviewers:</u>

We thank all three reviewers for carefully reporting on our manuscript. All are generally positive about the idea, content, rigor and presentation of our work and have certain points that we addressed in our revisions.

In particular, Reviewer #1 finds that our "work is sound and the subject interesting", judges that the "results are clearly exposed" and finds that "synchronization has not been studied in this specific [quantum setting]" and that "the work [is] technically sound".

Reviewer #2 is very positive about our work and emphasizes that the specific problem is only little "explored and is difficult to treat exactly, due in part to the exponential dimensiona[li]ty" and that the current work "makes a strong argument for the link between the synchronization transition in the analogous classical system, and the development of entanglement and squeezing in the quantum dynamics." The reviewer concludes that it would "provide[…] a significant contribution and focuses on an elegant system where robust connections can be made." and recommends the work for publication.

Reviewer #3 finds "the manuscript … well-crafted, the analysis … significant, and most importantly the research … novel." The reviewer emphasizes that our work "represents the first … such direct link between classical synchronization and quantum dynamics" and, as already Reviewer #2, also Reviewer #3 "recommend[s] publication in Nature Communications."

All reviewers have some questions and request a number of refinements of the work that we address on a point-by-point basis below, separately for each reviewer.

In particular, as suggested by two reviewers, the revised manuscript now provides additional data on the effect of dissipation in an additional subsection to the manuscript. It now clearly shows the robustness of our predictions with respect to opening the systems, realizing dissipation through phase noise.

<u>Replies to the comments of Reviewer #1</u>

We thank the reviewer for a thorough review of our manuscript.

1) After a brief summary of our main results, Reviewer #1 states that

"The work is sound and the subject interesting. There is some fragmentation in presenting general conclusions: indeed some of them are for N=2, some for large systems (L), some for BEC (with near neighbors couplings) and some others for all-to-all couplings… Nevertheless all results are clearly exposed in the main manuscript and several details are added in the Supplementary Information file. To the best of my knowledge synchronization has not been considered in this specific model."

Our Reply:

We thank the reviewer for carefully working through the manuscript and for these positive comments. We address the issues raised later below.

2) **The reviewer asks us to clarify the novel aspects of our work and to more closely embed them in previously published work on features of synchronization in quantum systems.**

"On the other hand, there have been several works on quantum synchronization and the authors do not properly refer to the state of the art on the subject.

The main advance of this work, as claimed by the authors, would be to show the "first" connection between synchronization and entanglement. This, however, is not a novelty and the authors should properly refer to previous works (not more specific than the particular case analyzed in this manuscript) where this question was already discussed. Reference [27], that is actually cited, also discusses the connection between synchronization and entanglement, showing that actually they do not always coincide. Furthermore, a relation between synchronization and quantum correlations or entanglement, is shown for instance, in Manzano et al, Sci. Rep. 3, 1439 (2013), Lee and Cross, PRA 88, 013834 (2013), Giorgi et al, PRA 88, 042115 (2013), Lee et al, PRE 89, 022913 (2014).... The results of this manuscript refer to a specific model where the threshold for entanglement and for synchronization coincide. Being a connection between these features already reported in some previous works, the authors should better identify the novelty and importance of their work with respect to the others."

Looking at other results of this work, the identification of a Kuramoto model for the dynamics of a quantum system in the classical limit is also not new. Indeed, both the mapping into Kuramoto models and its absence have been reported in the literature: see, for instance, the case of of opto-mechanical systems Heinrich et al. PRL 107, 043603 (2011), and Holmes et al., PRE 5, 066203 (2012), respectively.

Finally, the authors claim at page 9 that the absence of dissipation in this model, with respect to few other references they cite, represents an important advance. As a matter of fact, I do not find this to be really a key point. In general, non-linear dynamical systems with reciprocal coupling are susceptible to synchronize, and dissipation may be important or not in creating self-sustained oscillations. Synchronization can be studied in absence of dissipation, even in quantum systems, see for instance Liu et al., EPL, 103, 17007 (2013).

Furthermore, for the question addressed here by the authors, the absence of dissipation (and therefore of decoherence) and presence of non-linear couplings would naturally favors the presence of entanglement. So, I would actually find more interesting (or surprising) a connection between synchronization and entanglement in presence (and in spite) of dissipation."

Our reply:

We thank Reviewer #1 for pointing out important options for improvement, in particular with respect to the influence of dissipation and in the presentation and for pointing us to additional literature.

Regarding the question about the system class studied, we would like to stress that our investigation indeed holds for a broad class of Hamiltonian systems, such as the ones described by the Hamiltonian

of Eq. (1) in our paper. Such system Hamiltonians describe different kinds of coupled local oscillators, from nanomechanical oscillators to ultracold atoms in periodic lattice structures and beyond, demonstrating its range of applicability. In the revised manuscript, we have now added two additional references [16,17] on further potential experimental realizations.

We thank the Reviewer for pointing out several references concerned with synchronization in quantum contexts. We walk through the list suggested in detail:

a) Papers by R. Zambrini's group and by Lee et al.: All these papers deal with synchronization in dissipative systems. To the best of our knowledge, dissipation in these studies is crucial in reaching the synchronized state (sometimes also characterized by an enhancement of quantum correlations), so that synchronization is somehow induced by dissipation. Moreover, at least on the classical level, many scenarios are possible and synchronization may or may not occur.

In contrast, in the manuscript we report synchronization phenomena not induced by dissipation. Instead, the synchronization we predict and the corresponding enhancement of quantum correlations at the synchronization transition are intrinsic to the systems' own dynamics. As a consequence, we believe that the two issues are different.

This notwithstanding, we agree that it is interesting and relevant to study whether this intrinsic synchronization and the corresponding quantum effects survive in spite of dissipation. To address this question, we have therefore now added a dedicated section offering insights into the influence of dissipation on the synchronization phenomena we report. Please see below for more details.

b) Papers by the Marquardt group and by Homes/Meaney/Milburn: Whilst it is certainly true that there is a mapping in the classical limit onto oscillator models, all these models are dissipative from the start and hence it applies what is said in the previous point a). Furthermore, Heinrich et al focus on the dynamics in the classical limit, not on the quantum-classical correspondence. Homes et al do study quantum effects, yet their model is fundamentally different from the celebrated Kuramoto model. Indeed, the authors explicitly state that `Synchronization is lost via a mechanism involving a Hopf and heteroclinic bifurcation similar to that found in large amplitude forcing, rather than the sniper bifurcation that is involved in small amplitude forcing and, although in a reduced form, in Kuramoto's phase model.'

c) Paper by Liu et al.: This paper indeed considers classical synchronization and the question of what survives from it in the quantum realm in the absence of dissipation. They use, however, a wording "dephasing" which, as far as we see, refers to what are "coherent oscillations".

The main message of that paper is distinctly different from the one reported in our manuscript. Their results suggest that synchronization occurring in the classical systems is systematically degraded by quantum fluctuations. In contrast, our results show that the classical synchronization transition indicates the onset of dynamical growth of quantum fluctuations; it implies the creation of strong entanglement. Therefore, the observation the onset of synchronization in the form of phase locking offers a novel link to the onset of quantum correlations.

Moreover, Liu et al. do not refer to the same entanglement measures as we do, they state – only in the outlook – that "spin squeezing" could be further studied. This is exactly what we do since our entanglement measure is directly connected to quantum mechanical squeezing and quantum number

fluctuations. The strong entanglement we predict indeed is persistent, not destroyed by the quantum nature of the system. There is also no contradiction since at the point when strong entanglement is created our studied systems leave the manifold on which we have the mapping to the classical synchronization model (please see discussion between Eqs. (2) and (4)), hence it would be difficult to define synchronization at this stage on the quantum level.

Changes in response to the above points:

In the revised manuscript, we have more thoroughly worked out the motivation and presentation of the results in the light of previous work. In particular, we have added new references citing all papers mentioned by the reviewer in the revised version of the manuscript, see the new references 30, 31, 34, 36, 37, 39, 41 and 42. Even if these works focus on related and distinctly different aspects than our paper, this should give credit to the works and achievements mentioned by the Reviewer. We further note that exemplary papers have been cited in our original manuscript already, please see references 26-30 therein (references 32, 33, 35, 38 and 40 in the new version).

Concerning the question of the simultaneous presence of dissipation and entanglement, which is already studied in the first list of papers mentioned above. We agree that this question is very interesting to investigate in full detail. Strong dissipation may lead to situations in which subspaces exist which are not affected by the dissipation (so called dark states or decoherence-free subspaces). If those subspaces allow for synchronization and entanglement then both properties could obviously be found in such cases as well. As our work takes the perspective of non-dissipative systems, and now demonstrates robustness against dissipative perturbations, this realm of questions calls for a comprehensive new work, clearly going beyond the scope of our manuscript. We note that concerning only entanglement (without synchronization) much work has been done in related directions already, e.g., by the Zoller and the Cirac groups, as well as by some of us, see e.g. the review Eur. Phys. J. ST 224, pp. 2127-2171 (2015) and the references therein). In the revised manuscript, to directly link to the core statements of our manuscript, we added new numerical data and a corresponding new section that shows that our predictions are indeed robust to dissipation (as long as it is not too strong). Please see Figure 5 and the respective subsection text in the new version.

The reviewer criticizes that we would claim to demonstrate the "first" connection between synchronization and entanglement. This not the case and seems to be based on a misunderstanding. We actually never used the word “first”. Yet two statements could have been interpreted in this way, so we now removed them from the manuscript. Furthermore, in the revised manuscript, we discuss previous works in much more detail now, also contrasting existing links between synchronization and quantum dynamics to our advances, also compared to those discussed above.

As suggested by the reviewer, the revised manuscript now also provides evidence of robustness of our main findings with respect to dissipation as reflected in phase noise. In the discussion we further elaborate on the great potentials in ongoing experiments and stress that the current model class (in particular the specific system of Figure 3) provides an analytically solvable setting for the emergence of a strongly correlated phase in a quantum many-body system.

<u>Replies to the comments of Reviewer #2</u>

We thank the reviewer for a thorough review of the manuscript.

1) **Reviewer #2 summarizes the main contributions of our work:**
"This is a paper that studies a model quantum many-body system as described by the Hamiltonian presented in Equation 1 of the manuscript. The model contains a set of coupled boson oscillators, with intrinsic frequency, onsite (self) interactions, and a specific form of oscillator-oscillator coupling. It should be emphasized that the problem studied is that of an isolated quantum system (i.e. the dynamics that is considered is that for a system that does not contain dissipation)."

Our Reply:

We thank the reviewer for a careful consideration and for highlighting that dissipation is actually not of any principal issue in our work. As suggested by another reviewer, we now show the robustness of our results with respect to dissipation as realized by external phase noise, please see our comments below.

2) **The reviewer very positively judges the idea, our approach and the results and praises the presentation:**

"Firstly, let me comment on the topic. The main point is to make a connection between the extensively studied synchronization in networks of classical oscillators, with the problem of synchronization in quantum many-body physics. The lat[t]er is much less explored and is difficult to treat exactly, due in part to the exponential dimension[alit]y of the Hilbert space and the development of quantum entanglement and quantum correlations. This paper makes a strong argument for the link between the synchronization transition in the analogous classical system, and the development of entanglement and squeezing in the quantum dynamics.

Although this is certainly not the first time this connection has been explored, I believe the paper provides a significant contribution and focuses on an elegant system where robust connections can be made. The simplicity arises from the fact that the mean-field limit of the quantum system can be identified with a system of Kuramoto oscillators, which is the standard framework for approaching a description of classical synchronization. Furthermore, in the limit of two oscillators, the authors show that the quantum system reduces to a standard Lipkin-Meshkov-Glick model that may be familiar to some. I think it is particularly interesting to see the calculated behavior of quantum fluctuations around the critical phase locking points.

The paper is very well written, and I found it clear to follow, both the story and the mathematical details. The authors have paid considerable attention to the presentation. "

Our Reply:

We thank the reviewer for the positive comments about our paper. We address remaining questions and suggestions individually below.

2) **The reviewer in particular wonders if our conclusions of the system considered is not in complete isolation such that dissipation and noise play a role:**

"My main concern with this paper is really a fundamental one, and perhaps this is not a topic of criticism of the manuscript per se, but is important at a fundamental level. The absence of dissipation in this system means that the evolution is reversible Hamiltonian dynamics, and therefore the irreversible quantum synchronization that appears in the quantum dynamics of open quantum systems (for example lasers) is fundamentally different. It is known that the presence of dissipation rapidly leads to attenuation of entanglement and quantum correlations, so I wonder to what extent the conclusions of this paper rely on the fact that the system considered is completely isolated. This may affect the potential application of the results to a diverse set of physical systems that people consider."

Our reply:

The question raised by the Reviewer, in line with that of Reviewer #1, concerns the robustness of our predictions with respect to opening the systems and thus introducing dissipation. This aspect has been checked now and a corresponding new paragraph has been added, together with new data, please see also our reply to the last issue of Reviewer 1 above. Consequently, our predictions are shown to be robust in real-life experimental applications.

3) **The reviewer wonders about the usefulness of scaled units of time, energy and (thus) action.**

"My own personal preference is that hbar should not be set to one, as is done here, and that time and energy for example should have their usual dimensions."

Our Reply:

The reviewer touches an intricate point here. Obviously it would be good to use standard physical units, in particular for the time, to be able to compare result to a specific experiments. However, the systems studied in the present paper have intrinsic time scales given, e.g., by the oscillation period T=2*pi/omega (Figure 1) or the Bloch period T_B=2*pi/omega_B (Figure 4). From a theoretical view point, we believe that it is helpful to see the dynamics in relation to this intrinsic time scale and we thus use a normalized time, also enabling comparisons for different system settings that can be favorable also from an experimental point of view. For example, different groups use different atomic species (Rubidium, Lithium,…) in their experiments, so scaled units also provide advantages for experimentalists using the work.

To make them comparable, energy must be compared to the recoil energy and time to the respective intrinsic time scale in a given system. We have thus decided to keep the scaled units in the figures. A detailed discussion of how these parameters depend on the experimental settings, as e.g. the lattice depth, is given in the supplementary methods.

4) **The reviewer praises the presentation of our work and emphasizes the interest in the topic and by a broad field of researchers from distinct areas. In conclusions, the reviewer recommends publication.**

"The figures are reasonably clear, and a thorough list of references to previous work is provided. The authors consider a physical system in which the model could be implemented as a Bose quantum gas in a tilted optical lattice, looking at momentum space localization. I think this helps to make the discussion less abstract, and potentially could be of interest to AMO experimenters.

Overall, I think this is a well-written paper, addressing an interesting topic that is receiving considerable interest from a broad field of physicists who have backgrounds in traditionally distinct areas. I recommend it for publication."

Our Reply:

We thank the reviewer for the overall positive view of our work.

<u>**Replies to the comments of Reviewer #3**</u>

We thank the reviewer for a thorough review of our manuscript.

1) After a summary of the content of our manuscript and its perspectives, the reviewer praises the significance of our analysis, the novelty of the research as well as the presentation and recommend publication in Nature Communications.

"In their manuscript "Quantum signatures of synchronization" the authors study the dynamics of a many-bodied quantum Schrodinger equations. Remarkably, they draw a clear link between the quantum properties associated with the aforementioned system, including entanglement, coherence, squeezing, etc., and the synchronization dynamics of classical synchronization, specifically the Kumamoto model. In particular, they derive the equations of motion of the Kumamoto model directly from the Schrodinger equation and show that, for instance, entanglement in the quantum system emerges precisely at the onset of synchronization in the classical system. In addition to these theoretical results, the authors suggest experiments where their results may be observed (detailing the theoretical backing in the SM).

The manuscript is well-crafted, the analysis is significant, and most importantly the research is novel. Given that this work represents the first (to my knowledge) such direct link between classical synchronization and quantum dynamics, I recommend publication in Nature Communications."

Our reply:

We thank the reviewer for the overall very positive evaluation and recommendation to publish in Nature Communications. We address the remaining "small points" raised by the reviewer individually below.

2) "A few small points:

One of the most central links between the quantum and classical systems (i.e., Eqs. (1) and (3)) is the frequency terms \omega_l. Can the authors give a brief interpretation of their significance in Eq. (1) and the physical translation to Eq. (3). Moreover, in the example in the manuscript the authors use

Lorentzian distributed frequencies. Is this choice made for a specific reason? Does the Lorentzian itself have a physical meaning?"

Our reply:

We thank the reviewer for this important question. Interestingly, the correspondence between quantum and classical dynamics does not depend on the choice of the natural frequencies at all in the sense that the analysis holds for arbitrary fixed sets of N frequencies. Moreover, the analysis of the limit of large system size L is independent of this choice. We have used a Lorentzian distribution only for illustrative purposes in Figure 3. We have chosen a Lorentzian here because it is the standard example in the literature on the Kuramoto model and because the critical coupling K_c can be stated analytically in this case.

We agree, of course, completely with the reviewer that the precise impact of the actual distribution of frequencies is worthwhile to be studied in detail. Interestingly, this is possible for the types of experiments we propose based on ultracold atoms since there the local energy levels in the potential wells can be engineered largely at will e.g. by external laser fields, magnetic field gradients, gravity or by laser speckles superposed to the lattice structure. The latter case would be a realization of a random distribution of levels. We have added a paragraph in the Discussion section in which we now mention these experimental possibilities.

3) "From Eq. (3) to Eq. (6) the authors change the subscripts from l to n. Is there a reason for this. (I believe that my initial confusion came from assuming that l = 1,...,L and n = 1,...,N.)"

Our reply:

We thank the reviewer for the detailed and careful reading of the manuscript. There is no reason and we have removed this discrepancy to improve the clarity of the paper.

4) "A small correction: below Eq. (6) the authors state that phase-locking occurs when |\omega_n-UN/L| \le Kr, however this is not true if the mean <\omega_n-UN/L> is not zero. If the frequencies themselves are initially set to have mean zero, then since UN/L is a uniform shift for all oscillators then the criteria is just |\omega_n| \le Kr."

Our reply:

We thank the reviewer for this helpful comment. We have rewritten the respective sentence and also the Supplementary Information accordingly.

5) "In the case of the Bose-Einstein optical lattices the energies \omega_n, and thus the frequencies, are uniformly distributed, \omega_n=\omega_B*n. I am curious about the phase-transition to synchronization in the large system limit, where the distribution would be uniform. In the paper referred below it is shown that the phase-transition will be first-order. Does this have any physical significance?

D. Pazo, "Thermodynamic limit of the first-order phase transition in the Kuramoto model," Phys. Rev. E 72, 046211 (2005)."

Our reply:

As stated before, this does have a great significance for the synchronization transition, but not for the quantum-classical correspondence. We have added a paragraph in the discussion section where we address this point and cite the paper by Pazo (reference 43) and a review paper on the topic (reference 44).

Reviewer #1 (Remarks to the Author):

The resubmitted manuscript has been extended putting now this work into the context of previous results on quantum signatures of synchronization and showing the effect of dephasing on this isolated system.

In answer to the authors comments, my previous report assessed the strength and novelty of the submitted work in the context of quantum synchronization, avoiding vague judgments and referring to some of the papers published in the last years. With the due differences, those papers showed that some claims of novelty in the first version of this manuscript were not fair. There was not misunderstanding about this (as answered by the authors), as I was referring to claims like: "no direct correspondence between [synchronization and entanglement] is known" or "Despite the fundamental nature of synchronization and entanglement in classical and quantum systems, resp., no direct correspondence between them is established" appearing in the abstract and introduction.
These claims describing the state of the art were rather clear in my opinion and not correct, and have now been removed.

On the other hand, the resubmitted manuscript has been revised to explicitly acknowledge previous advances on quantum synchronization and actually it includes the context of references provided in my report, as a main part of the DISCUSSION section of the resubmitted manuscript. Indeed, there have been previous works on 'Quantum signatures of synchronization', related to different experimental platforms, and also in isolated systems. After reading the resubmitted manuscript and the authors answer about the novel results here and their importance, I still find that this work is incremental and does not reach the standards of Nature Communications.

Finally, I did not (mean to) suggest to study the effect of dephasing in this system. As I was not recommending the paper for publication, I did not suggest improvements or clarifications on any technical points. There was only a statement about the fact that finding coherence and entanglement in absence of decoherence is not so surprising as the authors were claiming. New plots in presence of dephasing noise address now decoherence and result are as expected: the presence of coupling (K) is clearly necessary to maintain coherence and decoherence is induced by a dephasing environment.

Reviewer #3 (Remarks to the Author):

In their revised manuscript the authors have sufficiently addressed each of my concerns and it seems to me those of the other referees as well. The paper is well-written, the presentation is clear, the analysis is sound, and most importantly the work itself is both novel and important. As a researcher in the area of nonlinear dynamics I particularly find the connection that this work makes to quantum physics fascinating, and I imagine that quantum physicists find the converse connection as fascinating. I believe that this work will be important and useful to scientists from a wide range of disciplines, and therefore I recommend publication in Nature Communications.

REPLIES TO REMAINING REVIEWER COMMENTS:

Reviewer #1 (Remarks to the Author):
The resubmitted manuscript has been extended putting now this work into the context of previous results on quantum signatures of synchronization and showing the effect of dephasing on this isolated system.

In answer to the authors comments, my previous report assessed the strength and novelty of the submitted work in the context of quantum synchronization, avoiding vague judgments and referring to some of the papers published in the last years. With the due differences, those papers showed that some claims of novelty in the first version of this manuscript were not fair. There was not misunderstanding about this (as answered by the authors), as I was referring to claims like:
"no direct correspondence between [synchronization and entanglement] is known" or "Despite the fundamental nature of synchronization and entanglement in classical and quantum systems, resp., no direct correspondence between them is established" appearing in the abstract and introduction.
These claims describing the state of the art were rather clear in my opinion and not correct, and have now been removed.

On the other hand, the resubmitted manuscript has been revised to explicitly acknowledge previous advances on quantum synchronization and actually it includes the context of references provided in my report, as a main part of the DISCUSSION section of the resubmitted manuscript. Indeed, there have been previous works on ‘Quantum signatures of synchronization’, related to different experimental platforms, and also in isolated systems. After reading the resubmitted manuscript and the authors answer about the novel results here and their importance, I still find that this work is incremental and does not reach the standards of Nature Communications.

Finally, I did not (mean to) suggest to study the effect of dephasing in this system. As I was not recommending the paper for publication, I did not suggest improvements or clarifications on any technical points. There was only a statement about the fact that finding coherence and entanglement in absence of decoherence is not so surprising as the authors were claiming. New plots in presence of dephasing noise address now decoherence and result are as expected: the presence of coupling (K) is clearly necessary to maintain coherence and decoherence is induced by a dephasing environment.

Our replies: We thank the reviewer for supplying another report. The work has been modified in accordance with the comments and questions of three reviewers in the previous round of review and the statements about novelty that were seen critically by reviewer #1 are not present in the current version. Indeed, with “direct” correspondence we meant a correspondence between persistent entanglement in isolated quantum systems and synchronization, as now clearly stated. Given the comments of two reviewers, including #1, it seemed to us an important question whether the phenomenon persists in the presence of dissipation, given also the statement of reviewer #1 that in isolated systems the phenomenon was not seen as “not surprising”. As stated in the previous round of review, to the best of our knowledge, synchronization in isolated quantum systems has not been found to induce persistent entanglement or systematically studied at all before. We believe that the revised version of our manuscript resubmitted previously indeed worked out the remaining issues.

Reviewer #3 (Remarks to the Author):

In their revised manuscript the authors have sufficiently addressed each of my concerns and it seems to me those of the other referees as well. The paper is well-written, the presentation is clear, the analysis is sound, and most importantly the work itself is both novel and important. As a researcher in the area of nonlinear dynamics I particularly find the connection that this work makes to quantum physics fascinating, and I imagine that quantum physicists find the converse connection as fascinating. I believe that this work will be important and useful to scientists from a wide range of disciplines, and therefore I recommend publication in Nature Communications.

Our replies: We again thank the referee for the positive judgement and for recommending the manuscript for publication in *Nature Communications*.

LIST OF CHANGES IN THE MAIN MANUSCRIPT:

- Changed the title according to the editor's suggestion
- Updated the references to the Supplementary material ("Supplementary Note", Arabic numbering)
- Checked math typesetting. All non-variable indices are set in upright roman font. In particular this applies to the index "B" in the Bloch frequency and the Bloch time and the index "c" for the critical coupling "K_c".
- Relayouted all figures: Use Helvetica Font only, Use Larger Fonts for better readability, Changed size and arrangement of Panels for better visibility.
- Revised the figure Captions. We have slightly extended the Figure Captions to make them more self-contained as suggested by the editor.
- Revised the Reference list: Added start-end page numbers for several journal articles. Added DOIs for all entries where available. Reformatted the Entries in Nature style.
- Removed Line numbering and typeset in two-column format.

LIST OF CHANGES IN THE SUPPLEMENTARIES:

- Removed the title page.
- Revised Figure Layout: All Panels are individually referenced by a letter a,b,… Increased size of figures for better visibility.
- Changed the Layout of the Section Headers: Different Font, Arabic instead of Roman Numbers.
- Revised the Reference List: Added start-end page numbers for several journal articles. Reformatted the Entries in Nature style.
- Removed Line numbering.
